# Secondary nucleotide messenger c-di-GMP exerts a global control on natural product biosynthesis in streptomycetes

**DOI:** 10.1093/nar/gkz1220

**Published:** 2020-01-20

**Authors:** Roman Makitrynskyy, Olga Tsypik, Desirèe Nuzzo, Thomas Paululat, David L Zechel, Andreas Bechthold

**Affiliations:** 1 Pharmaceutical Biology and Biotechnology, Institute of Pharmaceutical Sciences, Albert-Ludwigs University, Freiburg 79104, Germany; 2 Organic Chemistry, University of Siegen, Siegen 57068, Germany; 3 Department of Chemistry, Queen's University, Kingston, Ontario K7L 3N6, Canada

## Abstract

Cyclic dimeric 3′-5′ guanosine monophosphate, c-di-GMP, is a ubiquitous second messenger controlling diverse cellular processes in bacteria. In streptomycetes, c-di-GMP plays a crucial role in a complex morphological differentiation by modulating an activity of the pleiotropic regulator BldD. Here we report that c-di-GMP plays a key role in regulating secondary metabolite production in streptomycetes by altering the expression levels of *bldD*. Deletion of *cdgB* encoding a diguanylate cyclase in *Streptomycesghanaensis* reduced c-di-GMP levels and the production of the peptidoglycan glycosyltransferase inhibitor moenomycin A. In contrast to the *cdgB* mutant, inactivation of *rmdB*, encoding a phosphodiesterase for the c-di-GMP hydrolysis, positively correlated with the c-di-GMP and moenomycin A accumulation. Deletion of *bldD* adversely affected the synthesis of secondary metabolites in *S. ghanaensis*, including the production of moenomycin A. The *bldD*-deficient phenotype is partly mediated by an increase in expression of the pleiotropic regulatory gene *wblA*. Genetic and biochemical analyses demonstrate that a complex of c-di-GMP and BldD effectively represses transcription of *wblA*, thus preventing sporogenesis and sustaining antibiotic synthesis. These results show that manipulation of the expression of genes controlling c-di-GMP pool has the potential to improve antibiotic production as well as activate the expression of silent gene clusters.

## INTRODUCTION

One of the most remarkable features of streptomycetes is their ability to synthesize bioactive secondary metabolites (SMs). Genome sequencing has revealed that the genomes of streptomycetes typically contain 20–30 SM biosynthetic gene clusters (BGCs) (1–4). However, only a small number of these clusters are strongly expressed under laboratory conditions while the rest remain either poorly expressed or completely silent for unknown reasons. Many approaches have been used to activate the expression of cryptic clusters, including overexpression or inactivation of pleiotropic regulators, rational promoter engineering, heterologous expression and application of chemical elicitors (4–10).

Biosynthesis of SMs is tightly linked to morphological differentiation in streptomycetes. On solid media, streptomycetes typically exhibit the formation of pigmented spores and aerial mycelia. The progression of this morphological differentiation requires the activity of two different classes of genes (11). Genes that are involved in the formation of aerial mycelium are denoted *bld* (‘bald’) due to the glossy and wrinkled phenotype of strains with mutations in these genes. White (*whi*) mutants develop aerial hyphae in a normal way but they fail to complete differentiation in order to form mature chains of spores. In many cases, mutants with deletions in *bld* and *whi* genes are also deficient in SM production (12–19).

*Streptomyces ghanaensis* ATCC14672 is a producer of moenomycin A (MmA), a pentasaccharide antibiotic that belongs to a small family of phosphoglycolipids. MmA is considered a promising lead to combat vancomycin- and methicillin-resistant pathogens (20). MmA is the only known specific natural inhibitor of peptidoglycan glycosyltransferases involved in bacterial cell wall biosynthesis, making this compound an ideal candidate for the development of a novel class of antibiotics (21). As wild-type *S. ghanaensis* produces MmA in low quantity, studies on the regulation of its biosynthesis were initiated. Uncommonly for SM biosynthesis, the MmA BGC (*moe* cluster) does not include any cluster situated regulators (CSR) (20), and it was concluded that MmA production in *S. ghanaensis* is mainly controlled by pleiotropic regulators (22). It was found that AdpA_gh_, an *S. ghanaensis* ortholog of the *Streptomyces coelicolor* and *Streptomyces griseus* master regulator, directly activates transcription of *moe* genes. As well, it was shown that the translation of UUA-containing *adpA_gh_* mRNA, and other *moe* genes is dependent on the tRNA BldA_gh_. In this way AdpA and BldA form a feedback loop that positively influences their own expression (23). *WblA_gh_* was also shown to be involved in MmA regulation as mutant with a deletion in *wblA_gh_* overproduced MmA (24).

C-di-GMP is a key effector molecule in bacteria (25). In *S. coelicolor* the transcriptional regulator BldD controls the expression of *adpA* (also known as *bldH*), *bldA*, as well as dozens of other development-related genes (26). Interestingly, the regulatory activity of BldD is controlled by c-di-GMP. In the presence of c-di-GMP, a dimeric BldD complex bound to four c-di-GMP molecules is formed, which then proceeds to bind to target promoter sites (27). The biosynthesis and turnover of c-di-GMP is mediated by three different classes of enzymes. Biosynthesis is performed by diguanylate cyclases (DGCs), which condense two GTP molecules to form c-di-GMP using an active-site GGDEF domain. C-di-GMP can be degraded in two different ways by phosphodiesterases (PDEs). PDEs with an EAL domain hydrolyze c-di-GMP into linear pGpG molecules, which in turn spontaneously hydrolyze to form two molecules of GMP. In contrast, PDEs with a HD-GYP domain directly hydrolyze c-di-GMP to GMP. In many cases, domains with opposite activities are present in the same protein creating GGDEF-EAL or GGDEF-HD-GYP tandems. The tandem domain proteins mostly display one predominant activity, while the second domain remains inactive. C-di-GMP turnover domains often contain additional motifs important for ligand binding, allowing the cyclic dinucleotide molecule pool to be regulated in response to different environmental signals (25).

Although studies on the role of c-di-GMP in bacteria began more than 30 years ago (28), investigations on streptomycetes were initiated only recently with a main focus on morphological differentiation (26,29–30). Despite the fact that biosynthesis of SM is firmly coupled with morphogenesis, the role of c-di-GMP in the regulation of antibiotic production remained obscure. Recent studies demonstrated that overexpression of the genes *cdgA, cdgB, cdgC*, and *cdgD* encoding active DGCs in *S. coelicolor* severely altered the production of the blue-pigmented antibiotic actinorhodin (26,29,31), while the expression of an additional copy of *cdgB* in *Saccharopolyspora erythraea* increased erythromycin formation (32).

In this study, we investigated the role of c-di-GMP on MmA formation in *S. ghanaensis*. Overexpression of *cdgB_gh_* encoding a DGC increases the production of MmA, while deletion of *cdgB_gh_* has the opposite effect. Moreover, inactivation of *rmdB_gh_* encoding a PDE greatly increases MmA production, as well as activates the expression of several cryptic BGCs, including those encoding oxohygrolidin and desferrioxamine B biosynthesis. Deletion of *bldD_gh_*, encoding the key c-di-GMP modulated transcriptional regulator, essentially abolishes antibiotic biosynthesis. On combining different lines of evidence, we suggest that the observed phenotype of the *bldD_gh_* mutant is largely due to the strong upregulation of the *wblA_gh_* expression, which in turn represses secondary metabolism. Inactivation of the *rmdB_al_* specified PDE in *Streptomyces albus* is also observed to have an overall stimulatory effect on secondary metabolism, suggesting that a similar c-di-GMP-mediated regulatory network exists in other *Streptomyces* spp.. Overall, these results point to a broadly applicable strategy to improve antibiotic production and activate the expression of cryptic BGCs in actinomycetes that is based on manipulation of genes encoding c-di-GMP turnover.

## MATERIALS AND METHODS

### List of abbreviations and acronyms used in the work

A list of abbreviations and acronyms used in this work is given in Supplementary Note 1.

### Bacterial strains, media and culture conditions

Bacterial strains and plasmids used in this study are listed in Supplementary Table S1. All *Escherichia coli* strains were grown in Luria Bertani (LB) and 2 × YT media at 37°C supplemented with appropriate antibiotics if needed. Streptomycetes were cultured on soya flour mannitol agar (SFM), oatmeal agar, tryptic soy broth (TSB), SG and R5A media. *Streptomyces ghanaensis* was cultivated at 37°C and *S. albus* at 28°C on a rotary shaker at 180 r.p.m. Plasmids were introduced into *Streptomyces* strains by intergeneric conjugation with *E. coli* ET12567 (pUZ8002). Conjugations and selection of exconjugants were performed on SFM-agar supplemented with 60 mM CaCl_2_. The presence and stability of inheritance of integrative constructs in streptomycetes were checked as described earlier (33).

### Procedures for DNA manipulation

Routine cloning manipulations were made in *E. coli* XL1-Blue according to standard procedures (34). Oligonucleotides used in this work are listed in Supplementary Table S2. All enzymes were purchased from New England Biolabs. Polymerase chain reactions (PCRs) were performed using recombinant Phusion DNA polymerase (ThermoFisher). RedET-mediated gene replacements in plasmids were performed with the REDIRECT system (35). All constructs were verified by sequencing, PCR or restriction mapping.

### Gene deletions

To construct in-frame, marker-free deletions of *cdgB_gh_*, *rmdB_gh_*, *bldD_gh_* and *wblA_gh_* genes, the following general scheme was used. The gene of interest flanked with two homology arms (∼2 kb each) was amplified from the genomic DNA by PCR using an appropriate pair of primers. The resulting amplicon was cloned into EcoRV-digested pBluescriptKS+. Then the target gene was replaced by the *loxP*-flanked apramycin resistance cassette (*aac(3)IV*) from plasmid pLERECJ via recombineering. Next, *aac(3)IV* along with homology regions was PCR amplified with the same primer pair and subcloned into the hygromycin resistance (*hyg*) bearing suicide vector pKGLP2 cut with EcoRV. The final construct was conjugally transferred to *S. ghanaensis* with subsequent screening for apramycin resistant and hygromycin sensitive colonies (reflecting a double-crossover event and loss of the plasmid). The replacement of a gene in the *S. ghanaensis* chromosome was verified by PCR using a respective pair of primers. The Cre-expressing helper plasmid pUWLCre was then introduced into mutant to excise the apramycin gene from its genome.

To disrupt *XNR_1338* (*rmdB_al_*) in the *S. albus* chromosome, a 0.55 kb fragment carrying an internal part of the gene was amplified from genomic DNA by PCR with primers xnr_1338_vn_f and xnr_1338_vn_r. The resulting amplicon was cleaved with XbaI and EcoRV and cloned into respective sites of the suicide vector pKC1132 to give pKCXNR1338-vn. The latter was introduced into *S. albus* and selected for the resistance to apramycin (an indication of a single-crossover). The disruption of *XNR_1338* was confirmed by PCR (primers xnr1338_check and rmdB_EAL_rev; data not shown).

To inactivate the *pks3* and *fkbH* genes from the putative oxohygrolidin BGC, 1.1 and 0.5 kb long DNA fragments harboring an internal part of a gene were amplified from the genomic DNA by PCR with primers pks_vn_for and pks_vn_rev, and fkbH_RT_for and fkbH_RT_rev, respectively. The PCR products were digested with XbaI and EcoRV and then individually cloned into respective sites of pKC1132 to generate pKCpks-vn and pKCfkbH-vn. The resulting plasmids were further transferred into *S. ghanaensis* Δ*rmdB_gh_* and selected for the resistance to apramycin (an indicative of single crossover).

### Construction of plasmids for complementation and overexpression experiments

For the complementation experiments, 2.4, 2.8, 0.9 kb long DNA fragments containing *cdgB_gh_*, *rmdB_gh_* and *bldD_gh_* genes along with their putative promoter regions, respectively, were amplified from the genomic DNA by PCR with appropriate pair of primers. The obtained amplicons were treated with XbaI and EcoRI and then cloned into the XbaI/EcoRI-digested integrative vector pSET152 to create pSETcdgB, pSETrmdB and pSETbldD.

For the overexpression of *cdgB_gh_*, *rmdB_gh_* and *bldD_gh_* genes under *ermEp* control, 1.8, 2.5 and 0.56 kb DNA fragments, respectively, were PCR-synthesized using appropriate primers. For *cdgB_gh_* and *bldD_gh_* amplicons were cut with KpnI and EcoRV and ligated into KpnI/EcoRV-linearized pTES to give pTESacdgB and pTESabldD-expI. For *rmdB_gh_* PCR product was cleaved with EcoRI and ligated into EcoRV/EcoRI sites of pTES to afford pTESarmdB.

For the *rmdB_gh_* overexpression, a 2.8 kb long DNA fragment containing coding sequence along with its putative promoter region was amplified from the genomic DNA by PCR with appropriate primers. The PCR product was directly ligated into the EcoRV-cleaved vector pKC1139 resulting in pKCrmdB.

### Identification of BldD_gh_-binding sites

This was essentially done as described previously (23). Briefly, to identify conservative BldD-binding sites (BldDbs) in the *S. ghanaensis* genome, known BldDbs sequences were collected from GenBank. They were used as an input to search for the consensus motif with a help of the MEME software tool (36). Screening for the occurrence of identified motif within regions of interest was performed using FIMO software suite (37).

### Analysis of biosynthetic gene clusters

To identify the putative BGCs in the *S. ghanaensis* genome, antiSMASH v5.0 (38) was employed followed by manual annotation. The BLAST analysis was used to screen for orthologs in GenBank.

### Semiquantitative (sq)RT-PCR

Total RNA was isolated from *S. ghanaensis* grown for 48 h in TSB medium using RNeasy Mini Kit from Qiagen. To avoid DNA contaminations, RNA samples were treated with DNase I from NEB. Equal amount of total RNA from each studied strain (1 mcg in total) was used to synthesize cDNA using Photoscript II Reverse Transcriptase (NEB) according to instructions of the manufacturer. Two hundred nanograms of cDNA were used as a template for PCR reaction with appropriate primer pairs. As a control, primers specific to the sequence of *hrdB* encoding the RNA polymerase principal sigma factor were used. Negative controls were performed with *hrdB* specific primers in reactions without reverse transcriptase to confirm the absence of contaminating DNA in RNA samples. PCR products were separated on 1.5% agarose gels in TAE buffer and band intensity was evaluated with a help of ImageJ software. Total RNA samples were isolated from three independent biological replicates.

### Scanning electron microscopy

For scanning electron microscopy (SEM), small pieces of lawns were cut off SFM agar plates and directly analyzed on a Quanta 250 environmental scanning electron microscope (ThermoFisher).

### Construction of GusA reporter plasmids and β-glucuronidase activity measurements

Plasmids padpAscript, pmoeE5script and pbldAscript for the study of transcriptional activity of *adpA_gh_*, *moeE5* and *bldA_gh_* promoters, respectively, were constructed previously (23).

To probe the activity of *rmdB_gh_* promoter, a DNA fragment comprising 0.4 kb at the front of the translation start codon was amplified by PCR with appropriate primers. The *rmdB_gh_p* fragment was cloned into XbaI/KpnI-linearized pGUS to give prmdBscript. To evaluate the expression of *rmdB_gh_* on the translational level, a DNA region containing the entire stop codon-free gene along with putative promoter (400 bp upstream of the translation start codons) was amplified by PCR using an appropriate pair of primers. The amplicon was ligated to XbaI/EcoRV-cleaved pGUSHL4aadA, an integrative *Streptomyces* vector where the examined gene is fused to the *gusA* reporter gene through the helical peptide linker HL4, yielding prmdBtransl.

In order to substitute the TTA codon to CTG, PCR mutagenesis was applied to amplify an 8.2 kb DNA fragment from pSETrmdB with appropriate primers carrying a single codon TTA→CTG substitution. The obtained amplicon was treated with T4 Polynucleotide kinase and then self-ligated giving pSETrmdB-CTG. Next, a 2.7 kb DNA region comprising stop codon free *rmdB_gh_* along with its putative promoter region was PCR amplified and cloned into pGUSHL4aadA using the aforementioned procedure to create prmdB-CTGtransl. In control experiments, promoter free *rmdB_gh_*(TTA) and *rmdB_gh_*(CTG) genes without stop codon were amplified by PCR using respective set of primers and cloned into XbaI/EcoRV-digested pGUSHL4aadA, resulting in prmdBcontr and prmdB-CTGcontrol, respectively.

Measurement of β-glucuronidase activity was carried out as described previously (23). Cultures and subsequent β-glucuronidase assays were performed in triplicate. Values were normalized to equal amounts of dry biomass (10 mg) and presented as the mean ±2 standard deviations.

### Analysis of moenomycins production

Cultivation of *S. ghanaensis*, extraction, disc diffusion assay, and analysis of moenomycins was done by following the previously established protocol (22). Two compounds were monitored via LC-MS in *S. ghanaensis* extracts: MmA (*m/z* = 1580.6 [M-H]^–^) and nosokomycin B (NoB; *m/z* = 1484.6 [M-H]^–^). The mixture of these two dominant compounds is referred to as moenomycin in this work, and the mean value of their LC-MS peak area in *S. ghanaensis* ATCC14672 was taken as 100%. Amounts of moenomycin were normalized to equal amounts of biomass (dry weight) and were mean values from at least three independent biological repeats. LC-MS analysis was performed on a UHPLC Thermo Fisher Scientific Ultimate 3000 SD system equipped with an automated liquid sampler, a diode array detector, and a TSQ Quantum Access MAX ESI mass spectrometer with a reversed-phase Nucleodur 100–5 C18ec column (Macherey-Nagel, 5 μm, 150 × 2 mm). The mobile phase A: water and mobile phase B: MeCN, both with 0.5% acetic acid (vol/vol) as a solvent modifier. The solvents were delivered at 0.5 ml/min under a gradient elution program: 0 min 95% A, 0.5 min 95% A, 10.5 min 5% A, 12.5 min 5% A, 12.7 min 95% A, 15 min 95% A. The mass spectrometer was operated in negative ESI mode, with an ion-spray voltage of −3.5 kV, source temperature of 350°C. The pressures of sheath and auxiliary gas (N_2_) were set to 25 and 5 (arbitrary units) respectively. The ion transfer tube was heated up to 300°C.

### Analysis of SM produced by *S. ghanaensis* and *S. albus* strains

One-hundred-milliliter flasks with 15 ml of seed medium (TSB) were inoculated with equal amounts of spores (2 × 10^5^ cfu). After two days of cultivation one ml of resulting seed broth was used to inoculate 300 ml flasks with 50 ml of fermentation medium (TSB for *S. ghanaensis* strains and R5A for *S. albus*) with a subsequent cultivation for 5 days. The fermentation broth was spun down at 5000 r.p.m. for 10 min and then the supernatant was extracted with an equal volume of *n*-butanol or ethyl acetate. The organic phase was evaporated *in vacuo*, resuspended in MeOH, then filtered through a 0.2 μm PVDF filter. Next, the *S. ghanaenis* extracts were used for HPLC-MS analysis on an Agilent 1100 LC system connected to a G1946D mass spectrometer with a reversed-phase Xbridge C18 column (Waters, 3.5 μm, 100 × 4.6 mm) using water as mobile phase A and MeCN as mobile phase B, both containing 0.5% acetic acid (vol/vol) as a solvent modifier. Elution was carried out at 0.6 mL/min as follows: 0 min 95% A, 0.5 min 95% A, 18.5 min 5% A, 20.5 min 5% A, 20.8 min 95% A, 25 min 95% A. Full-scan mass spectra (*m/z* 200–2000) were collected in both positive and negative ESI modes. The following parameters were used: capillary voltage, 3000 V; nebulizer gas pressure, 35 psi; drying gas flow rate (N_2_), 10 l/min; drying gas temperature, 350°C.

Extracts from *S. albus* were analyzed on a UHPLC Thermo Fisher Scientific Ultimate 3000 SD system joined with a TSQ Quantum Access MAX ESI mass spectrometer. For the LC analysis, a Zorbax Eclipse Plus C18 column (Agilent, 1.8 μm, 50 × 2.1 mm) was used (mobile phase A: water and mobile phase B: MeCN, both with 0.5% acetic acid (vol/vol) as a solvent modifier). The solvents were delivered at 0.5 ml/min under a gradient elution program: 0 min 90% A, 0.5 min 90% A, 8 min 5% A, 9 min 5% A, 9.1 min 90% A, 10 min 90% A. Full-scan mass spectra (*m/z* 200–1500) were collected in both positive and negative ESI modes, with an ion-spray voltage of 2500 V, ion source temperature of 450°C. The pressures of sheath and auxiliary gas (N_2_) were set to 25 and 5 (arbitrary units), respectively. The ion transfer tube was heated up to 320°C.

MS/MS analysis of samples containing desferrioxamine B was performed on a UHPLC Thermo Fisher Scientific Ultimate 3000 SD system joined with a TSQ Quantum Access MAX ESI mass spectrometer with a Zorbax Eclipse Plus C18 column (Agilent, 1.8 μm, 50 × 2.1 mm; mobile phase A: water and mobile phase B: MeCN, both with 0.5% acetic acid (vol/vol) as a solvent modifier). The mass spectrometer was operated as described previously (39).

For high-resolution (HRMS) LC-MS, samples were measured on a Thermo LTQ Orbitrap XL mass spectrometer coupled to a UHPLC Thermo Dionex Ultimate 3000 RS. Analytes were separated on ACQUITY BEH C18 Column (Waters, 1.7 μm, 100 mm × 2.1 mm) with water + 0,1% formic acid (vol/vol) (mobile phase A) and MeCN + 0,1% formic acid (vol/vol) (mobile phase B). The following gradient was used: 0 min 95% A, 0.27 min 95% A, 18.27 min 5% A, 20.27 5% A, 21.27 95% A, 23.77 95% A with a flow of 0.55 ml/min.

### Large-scale growth of *S. ghanaensis*, purification and structure elucidation of oxohygrolidin

Large-scale production was done through a similar way to that for the small-scale fermentations described above. Two ml of two-days *S. ghanaensis* Δ*rmdB_gh_* seed culture prepared as described above were used to inoculate 500 ml flasks with 100 ml of TSB fermentation medium. In total, 4 l of fermentation culture was used for compound isolation. After 5 days of incubation the broth was centrifuged at 5000 r.p.m. for 20 min to separate a mycelium cake from a supernatant. The biomass was washed twice with distilled water. Oxohygrolidin was extracted by stirring the biomass with MeOH/water (9:1, vol/vol) at 37°C overnight. After centrifugation the supernatant was dried *in vacuo* ant then dissolved in distilled water. The compound was then extracted twice with an equal volume of ethyl acetate. The combined organic phase was dried *in vacuo*, resuspended in 40% MeOH, and fractionated using solid phase extraction column (Oasis HLB 35cc (6 g) LP Extraction Cartridge, Waters) with subsequent gradient elution with increasing concentrations of MeOH. The fractions containing the compound were concentrated *in vacuo* and further purified by semi-preparative LC on an Agilent 1100 HPLC system with a DAD detector equipped with a Zorbax SB C18 column (Agilent, 5 μm, 9.4 × 150 mm). The following elution program was used: 0 min 30% A, 0.5 min 30% A, 15 min 5% A, 17 min 5% A, 17.2 min 30% A, 20 min 30% A (mobile phase A: water and mobile phase B: MeCN). The solvents were delivered at 2.5 ml/min. This procedure yielded 8 mg of oxohygrolidin.

For determination of oxohygrolidin structure, mass spectra were recorded on a Thermo LTQ Orbitrap XL ESI mass spectrometer as described above. Nuclear magnetic resonance spectra (NMR) were measured on a Varian VNMR-S 600MHz spectrometer equipped with 3 mm triple resonance inverse and 3 mm dual broadband probe heads at 25°C. Spectra were recorded in 150 μl CD_3_OD. Residual signals were used as internal standard (δH = 3.30 ppm, δC = 49.0 ppm). The structure elucidation was done based on MS and 1D and 2D NMR spectra. Comparison to literature confirmed this compound to be oxohygrolidin (40).

### Protein production and purification

To probe catalytic activities of RmdB_gh_, truncated versions of the protein missing the transmembrane domain were produced. PDE (amino acid residues 432–763), DGC-PDE (amino acid residues 317–763) and DGC (amino acid residues 317–484) domains encoding sequences were cloned into pET28a resulting in *N*-terminally His_6_ tagged proteins. Extended versions of diguanylate cyclase domain including one transmembrane helix were cloned into pET24b and produced as C-terminally His_6_ tagged DGC-274 (amino acid residues 274–484) and DGC-302 (amino acid residues 302–484). *Escherichia coli* BL21 (DE3) Star carrying expression plasmid was grown in LB supplemented with kanamycin (50 μg/ml) until OD_600_ reached 0.5. Protein production was induced by IPTG (0.25 mM) and culture was then allowed to grow at 18°C for another 16–18 h. Cells were harvested by centrifugation and lysed in Tris-buffer (50 mM Tris-HCl, 0.5 M NaCl, 2 M urea, 5 mM imidazole, 10 mM PMSF and DNase I, pH 7.5) by passage through a French press. Proteins were purified by Ni-NTA affinity chromatography from a soluble fraction and eluted by increasing concentrations of imidazole (20, 50 and 250 mM). Fractions containing DGC, PDE or DGC-PDE were pooled and then dialyzed against storage buffer (50 mM Tris-HCl, 0.5 M NaCl, 5% glycerol, pH 7.5).

A recombinant BldD_gh_ was produced as *N*-terminally His_6_ tagged protein. For this purpose, its ORF was amplified from *S. ghanaensis* chromosomal DNA, digested with NdeI and XhoI restriction endonucleases and cloned into respective sites of pET28a, giving pET28a-bldD_gh_. After confirmation by restriction analysis and sequencing, the obtained plasmid was introduced into BL21 (DE3) Star where protein production was achieved by inducing the growing cells with 0.25 mM IPTG when optical density reached 0.5. Cells were collected after 16 h growing at 20°C in LB medium. Protein was purified by Ni-NTA chromatography in a buffer containing 50 mM Tris-HCl, 0.3 M NaCl and 5% glycerol, pH 8.0 with increasing imidazole concentrations (20–250 mM). The concentration of purified protein was determined by Bradford assay.

For YdeH production, its ORF was amplified by colony PCR from XL1Blue cells and cloned into NcoI and XhoI sites of pET28a. The recombinant protein was produced with *C*-terminal His_6_ tag as described before (41).

### *In vitro* enzymatic assay

The c-di-GMP synthesis activity of recombinant proteins was assayed in reaction mixture (100 μl) containing 50 mM Tris-HCl (pH 8.0), 50 mM NaCl, 200 μM GTP, 5 mM MgCl_2_ or MnCl_2_. The reaction was started by adding 5 μM (or 10 μM) of purified DGC and incubated at 37°C for 1 h. A control reaction using 2 μM YdeH (a DGC from *E. coli*) was performed identically.

A PDE activity assay was carried out in a DGC reaction buffer containing 200 μM c-di-GMP instead of GTP and 5 μM of PDE or DGC-PDE. After incubation at 37°C for 1 h, reaction was terminated by heat inactivation at 75°C for 10 min in a presence of 10 mM CaCl_2_, centrifuged at 14 000 r.p.m. for 10 min, and filtered through 0.2 μm PVDF filter. Analytes were measured on an Agilent 1100 HPLC system connected to a G1946D mass spectrometer with a Zorbax RX C8 column (Agilent, 5 μm, 4.6 × 250 mm) using solvent A: water with 10 mM tributylamine and 15 mM acetic acid and solvent B: MeCN. The following elution program was used: 0 min 95% A, 3 min 95% A, 7 min 70% A, 9 min 70% A, 20 min 5% A, 25 min 5% A, 25.5 min 95% A, 30 min 95% A with a flow of 0.5 ml/min. Full-scan mass spectra (m/z 300–750) were collected in negative ESI mode. The following parameters were used: capillary voltage, 3000 V; nebulizer gas pressure, 35 psi; drying gas flow rate (N_2_), 10 l/min; drying gas temperature, 350°C. C-di-GMP and GTP standards were purchased from InvivoGen and Sigma-Aldrich, respectively.

### Electrophoretic mobility shift assay

Promoter regions of *cdgB_gh_*, *rmdB_gh_* and *wblA_gh_* were produced by PCR from *S. ghanaensis* chromosomal DNA and then 5′ end-labeled using [γ^33^P]-ATP and T4 polynucleotide kinase. Twenty fmol of labeled DNA probe was incubated with purified BldD_gh_ at 37°C for 25 min in 15 μl binding buffer (10 mM Tris pH 7.5, 1 mM EDTA, 5% glycerol, 10 mM NaCl, 1 mM MgCl_2_) containing 1 μg poly(dI-dC) and 1 μM c-di-GMP. Also, increasing concentration of c-di-GMP (0.5–3 μM) were tested in reaction with *wblA_gh_p* and 0.75 μM BldD_gh_. The DNA–protein complexes were separated on 8% nondenaturing polyacrylamide gels in Tris-borate-EDTA buffer. After drying the gels, bands were visualized by phosphor imaging.

Competition assay was carried out in reaction sample containing 20 fmol labeled *wblA_gh_p* promoter incubated with 0.75 μM BldD_gh_, 1.5 μM c-di-GMP and 10-, 50-, 100- and 200-fold molar excess of unlabeled probe in binding buffer as described above.

### Intracellular c-di-GMP extraction and measurements

Streptomycetes samples were collected after 24, 48, 72 and 96 h of growth in TSB medium. Intracellular c-di-GMP was extracted with 40% methanol–40% acetonitrile in 0.1 N formic acid as previously described (42). The samples were analyzed on a UHPLC Thermo Fisher Scientific Ultimate 3000 SD system joined with a TSQ Quantum Access MAX ESI mass spectrometer. For the LC analysis, a Pursuit 3 PFP column (Agilent, 3 μm, 100 × 2 mm) was used (mobile phase A: water and mobile phase B: MeCN, both with 0.5% acetic acid (vol/vol) as a solvent modifier). The solvents were delivered at 0.5 ml/min under a gradient elution program: 0 min 97% A, 3 min 97% A, 5 min 5% A, 7 min 5% A, 7.3 min 97% A, 10 min 97% A. C-di-GMP was detected by MS/MS multiple reaction monitoring in negative ESI mode with an ion-spray voltage of 2500 V, ion source temperature of 350°C. The pressures of sheath and auxiliary gas (N_2_) were set to 65 and 17 (arbitrary units), respectively. The ion transfer tube was heated up to 255°C. The *m/z* 689.1→344.5 transition was used for quantitation while the 689.1→151.2 fragmentation was monitored as confirmatory signal. The collision energies were 36 and 46 eV, respectively. For a standard curve, 6.25, 12.5, 25, 50, 100, 200 and 500 nmol/l pure c-di-GMP (InvivoGen) dissolved in extraction buffer were analyzed by the above method. Values represent the mean of three independent biological replicates ±2 standard deviations.

## RESULTS

### c-di-GMP governs MmA biosynthesis in *S. ghanaensis*

**Figure 1.**
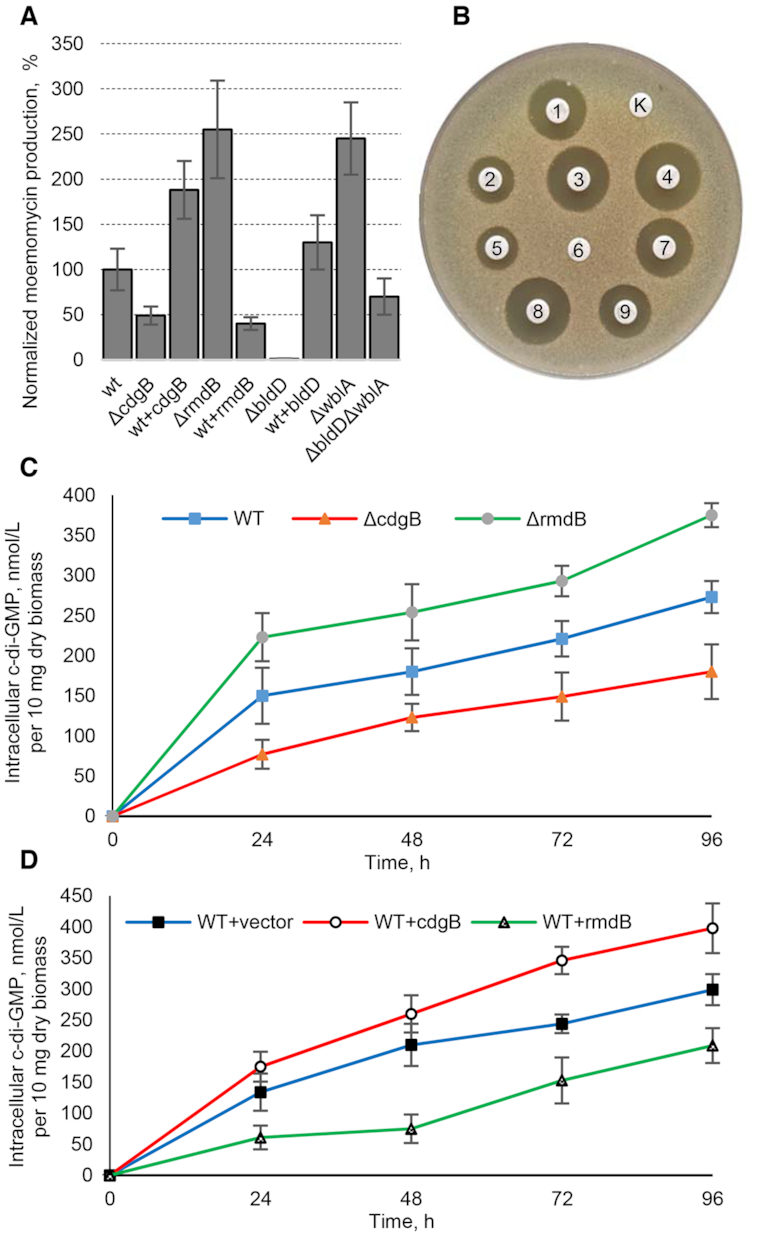
Levels of moenomycin and c-di-GMP production by various *Streptomyces ghanaensis* strains. (**A**) Moenomycin production titers by different *S. ghanaensis* strains studied in this work as determined by HPLC-MS. The mean value of moenomycin mass peak area in *S. ghanaensis* ATCC14672 was taken as 100%. Amounts of moenomycin were normalized to equal amounts of biomass (dry weight) and were mean values from at least three independent biological replicates. Error bars, ±2 SD. (**B**) *Bacillus cereus* growth inhibition around paper discs saturated with methanol extracts from equal amount of biomass of different MmA producers: *S. ghanaensis* ATCC14672 (1), Δ*cdgB_gh_* (2), ATCC14672 overexpressing *cdgB_gh_* (3), Δ*rmdB_gh_* (4), ATCC14672 overexpressing *rmdB_gh_* (5), Δ*bldD_gh_* (6), ATCC14672 overexpressing *bldD_gh_* (7), Δ*wblA_gh_* (8), Δ*bldD_gh_*Δ*wblA_gh_* (9), negative control, methanol (K). Intracellular c-di-GMP levels in the *S. ghanaensis* strains with gene deletions (**C**) and overexpressions (**D**). c-di-GMP was extracted at different time points from cells grown in TSB medium. Data represent means of three independent cultures and were normalized to equal amounts of dry biomass. Error bars are the standard deviations between independent cultures.

The *S. ghanaensis* genome encodes nine proteins putatively involved in c-di-GMP metabolism (Supplementary Table S3). To gain insight into the role of c-di-GMP in the regulation of antibiotic production, we decided to individually inactivate *cdgB_gh_ (ssfg_03956)* and *rmdB_gh_ (ssfg_02196)*, two genes predicted to code for enzymes with c-di-GMP synthesis and hydrolytic activities, respectively. Orthologs of these two genes are broadly conserved in streptomycetes and, in *Streptomyces venezuelae*, they have been shown to be expressed at high levels throughout the entire life cycle among all other genes encoding c-di-GMP turnover proteins (43). CdgB_gh_ contains a GGDEF catalytic domain that is found in DGCs and thus was chosen for further study. Deletion of *cdgB_gh_* reduced MmA production 2-fold, as determined by HPLC-MS and bioassays (Figure 1A and B). The Δ*cdgB_gh_* mutant also showed changes in morphogenesis by displaying an early onset into sporogenesis (Supplementary Figure S1). The integration of a native copy of *cdgB_gh_* into the *attB^φ^^C31^* site of the mutant restored MmA production and wild-type morphology in contrast to a strain carrying an empty vector. Introduction of an extra copy of *cdgB_gh_* under the control of constitutive promoter *ermEp* led to a 2-fold increase in MmA production (Figure 1A and B) while strongly inhibiting spore formation (Supplementary Figure S1).

In contrast to CdgB_gh,_ RmdB_gh_ contains both GGDEF- and EAL- domains. However, *in vitro* this enzyme displays only PDE activity (Supplementary Figure S2). Moreover, unlike the Δ*cdgB_gh_* mutant, Δ*rmdB_gh_* produced 2.5-fold more MmA (Figure 1). This mutant was also affected in morphological differentiation. Specifically, the aerial mycelium of the Δ*rmdB_gh_* mutant remained white even after prolonged incubation, in contrast to the dark-green colonies of the wild-type strain (Supplementary Figure S3A). Scanning electron microscopy revealed long aerial hyphae lacking chains of spores in contrast to normally developed spores in wild-type (Supplementary Figure S3B). The integration of a native copy of *rmdB_gh_* into the mutant restored both MmA production and morphogenesis in comparison to a control strain bearing an empty vector. Overexpression of *rmdB_gh_* resulted in a strain that produces only a small amount of MmA (Figure 1A and B). In this strain the timing of spore formation was accelerated (Supplementary Figure S3C).

To evaluate the role of BldD, the well-known sensor of c-di-GMP in streptomycetes, we generated a Δ*bldD_gh_* mutant. Production of MmA was strongly reduced in this strain (Figure 1A and B). As well, the Δ*bldD_gh_* mutant exhibited altered morphological development (Supplementary Figure S4). Scanning electron microscopy showed that BldD influences the timing of morphogenesis, especially the timing of sporulation. In contrast to *S. coelicolor* and *S. venezuelae* (16,27), in the Δ*bldD_g__h_* mutant, sporulation was clearly delayed (Supplementary Figure S4B and C). Complementation of the mutant with a native copy of the *bldD_gh_* gene restored morphological development and led to an increase in MmA production.

Next, we compared phenotypic differences in bacterial growth between the wild-type and mutant strains (Supplementary Figure S5A and B). Deletion of *cdgB_gh_* slightly decreased biomass accumulation, while the most prominent changes were observed in the Δ*bldD_g__h_* strain, which accumulated much lower amounts of biomass during first 48 h of growth (Supplementary Figure S5A).

We also assessed the changes in intracellular c-di-GMP levels in the *S. ghanaensis* mutants. Deletion of *cdgB_gh_* led to the reduction in the nucleotide messenger pool, while its overexpression positively correlated with the level (Figure 1C and D). In contrast to Δ*cdgB_gh_*, the Δ*rmdB_gh_* mutant accumulated 2-fold more of c-di-GMP. It was also found that, compared with the control strain carrying an empty vector, overexpression of *rmdB_gh_* decreased intracellular c-di-GMP concentration (Figure 1C and D).

### Impact of mutations in *rmdB_gh_, cdgB_gh_*, and *bldD_gh_* on the expression of genes involved in MmA biosynthesis and morphological differentiation

**Figure 2.**
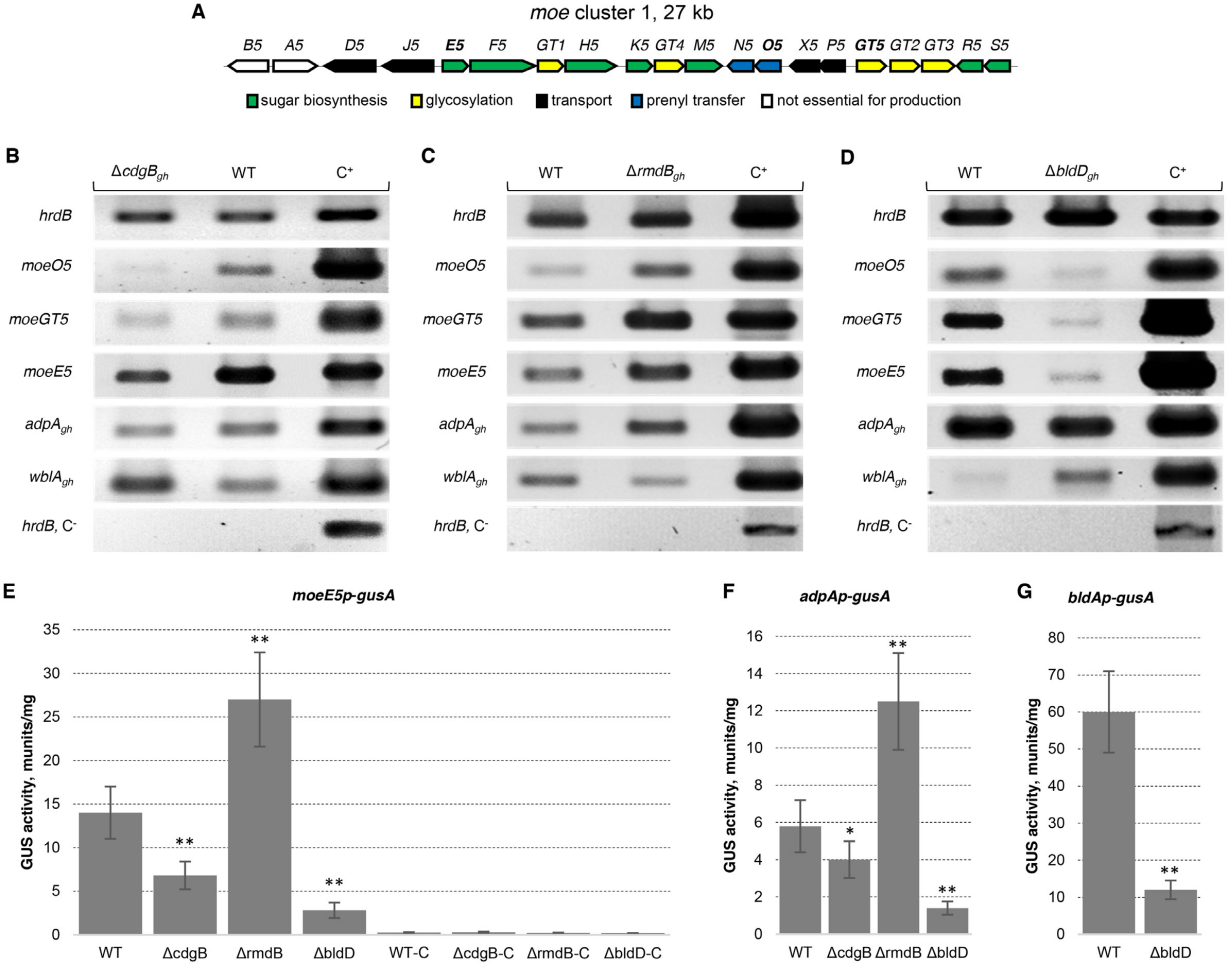
Deletion of *cdgB_gh_*_,_*rmdB_gh_* and *bldD_gh_* strongly influences expression of genes essential for moenomycin production. (**A**) Genetic organization of *moe* cluster 1. (**B**) Comparison of Δ*cdgB_gh_* and ATCC14672 transcriptional profiles. (**C**) Comparison of Δ*rmdB_gh_* and ATCC14672 transcriptional profiles. (**D**) Comparison of Δ*bldD_gh_* and ATCC14672 transcriptional profiles. The expression of tested genes was analyzed in 48 h cultures grown in TSB; 200 ng of RNA sample were used per reaction; C^+^, positive control (genomic DNA of ATCC14672 strain). Attempts to synthesize *hrdB* from RNA without pretreatment with RT served as negative controls (marked as C^−^). Total RNA samples were isolated from three independent biological replicates. The images represent the typical result of three independent RT-PCR experiments. (**E**) Transcriptional activity of the *moeE5* promoter in *Streptomyces ghanaensis* strains. (**F**) Transcriptional activity of the *adpA_gh_* promoter in *S. ghanaensis* strains. (**G**) Transcriptional activity of the *bldA_gh_* promoter in *S. ghanaensis* strains. WT-C, ΔcdgB-C, ΔrmdB-C and ΔbldD-C: control strains carrying promoterless vector pGUS. Cultures and subsequent β-glucuronidase assays were performed in triplicate. Values were normalized to equal amounts of dry biomass. Error bars, ±2 SD. Significance of difference in the transcriptional activities of tested promoters was calculated by a two-tailed t-test. Asterisks indicate the significance value (^∗^*P* < 0.05, ^∗∗^*P* < 0.01).

The influence of mutations in *cdgB_gh_, rmdB_gh_*, and *bldD_gh_* on the expression of genes (*moeO5, moeE5*, *moeGT5*) involved in indispensable steps of MmA biosynthesis (Figure 2A) (21) and on regulatory genes (*adpA_gh_* and *bldA_gh_*) was analyzed by sqRT-PCR and a GusA transcriptional reporter assay (44). As shown in Figure 2B, transcription of all *moe* genes was repressed in the Δ*cdgB_gh_* mutant. Likewise, the GusA reporter assay revealed more than 2-fold decrease in transcriptional activity of the *moeE5* promoter (Figure 2E). In contrast, deletion of *rmdB_gh_* elevated the expression levels of all three structural genes and *adpA_gh_* in comparison to the wild-type strain. The GusA reporter system confirmed a 2-fold increase in the transcriptional activity of the *moeE5* promoter (Figure 2C, E and F). However, deletion of *bldD_gh_* drastically repressed the transcription of *moe* genes (Figure 2D). In contrast to the results obtained previously (26), we also observed that *adpA_gh_* was less transcribed in the Δ*bldD_gh_* mutant than in the wild-type strain (Figure 2D). As indicated by the GusA reporter assay, the transcriptional activities of the *moeE5*- and *adpA_gh_*- promoters were reduced 5- and 4-fold, respectively (Figure 2E and F). In the Δ*bldD_gh_* mutant, transcriptional activity of the *bldA_gh_* promoter was decreased 5-fold (Figure 2G). This indicates that inactivation of *bldD_gh_* leads to an overall repression of MmA biosynthesis in *S. ghanaensis*.

To gain further regulatory insight into the Δ*bldD_gh_*-mediated phenotype in *S. ghanaensis* and following the idea put forward by den Hengst *et al.* (26), we examined the expression levels of putative regulatory genes *ssfg_03791*, *ssfg_03212* and *ssfg_03279* encoding a sporulation associated protein, a putative regulatory protein and a WhiB-family transcriptional regulator, respectively, which we hypothesized might be controlled by BldD_gh_. Only the expression of *ssfg_03279* was greatly enhanced in *S. ghanaensis* Δ*bldD_gh_* compared to the wild-type strain (Figure 2D). Gene *ssfg_03279* (*wblA_gh_*), which encodes an ortholog of pleiotropic regulator WblA of *S. coelicolor*, was previously shown to negatively affect MmA production and morphogenesis in *S. ghanaensis* (24). Interestingly, transcription of *wblA_gh_* was upregulated in *S. ghanaensis* Δ*cdgB_gh_* and downregulated in *S. ghanaensis* Δ*rmdB_gh_*, in comparison to the wild-type strain (Figure 2B and C).

### Deletion of *wblA_gh_* suppresses the Δ*bldD_gh_*-mediated phenotype in *S. ghanaensis*

**Figure 3.**
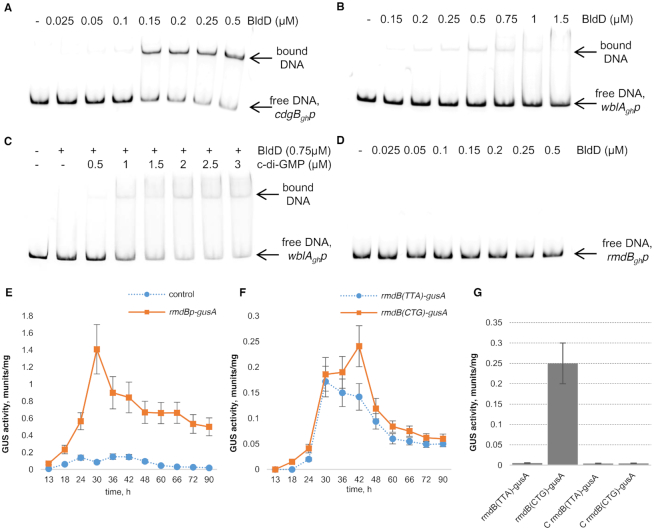
BldD_gh_ regulates the expression of *cdgB_gh_*, *wblA_gh_* and *rmdB_gh_*. EMSA analysis of BldD_gh_ binding to the *cdgB_gh_*(**A**), *wblA_gh_* (**B** and **C**), and *rmdB_gh_* (**D**) promoters. Twenty fmol of ^33^P-labeled probe was incubated with increased concentration of BldD_gh._ The reactions in A, B and D contained 1 μM of c-di-GMP. C-di-GMP concentrations used in (C) are indicated above each line. (**E**) Time dependency of the transcriptional activity of the *rmdB_gh_* promoter. (**F**) Translation efficiency of *rmdB_gh_* is regulated by the presence of the TTA codon. (**G**) Translation of *rmdB_gh_*(TTA) is strongly repressed in *Streptomycesghanaensis* Δ*bldA_gh_*. C rmdB(TTA)-gusA and C rmdB(CTG)-gusA correspond to promoterless fusions. Cultures and subsequent β-glucuronidase assays were performed from three independent biological replicates. Error bars, ±2 SD.

Previous work on transcriptional analysis of BldD-targeted genes in non-*Streptomyces* actinobacterium *Actinoplanes missouriensis* revealed that BldD represses dozen genes, including a *wblA* ortholog (45). A mutant of *S. ghanaensis* lacking *wblA_gh_* accumulated approximately 2.5-fold more MmA than the wild-type strain (Figure 1A and B). It also displayed a ‘white’ phenotype, indicating that spore maturation was inhibited (Supplementary Figure S6A). We thus proposed that the strong abolishment of MmA production in Δ*bldD_gh_* is due to the high expression of *wblA_gh_* that would repress antibiotic biosynthesis. To address this hypothesis, the double mutant *S. ghanaensis* Δ*bldD_gh_*Δ*wblA_gh_* was generated. This mutant restored the capacity to produce MmA (Figure 1A and B), indicating that WblA_gh_ plays a crucial role in the regulation of MmA biosynthesis and that BldD_gh_ controls the expression of *wblA_gh_*. Direct control of the *wblA_gh_* transcription by BldD_gh_ was further supported by electrophoretic mobility shift assay (EMSA) with purified BldD_gh_ (see below, Figure 3B and C). We also verified this hypothesis by overexpressing *cdgB* from *S. coelicolor* (29) (plasmid pIJ10350) in *S. ghanaensis* Δ*wblA_gh_* as well as in the wild-type strain. In contrast to the wild-type strain carrying pIJ10350, where antibiotic synthesis was strongly uplifted, MmA production was not further improved by the expression of the gene in Δ*wblA_gh_* (Supplementary Figure S7). It is noteworthy that expression of *adpA_gh_* remained unaffected in *S. ghanaensis* Δ*wblA_gh_* in comparison to the wild-type strain (Supplementary Figure S8), indicating that WblA_gh_-depended phenotype is likely not mediated by AdpA_gh_. Finally, *S. ghanaensis* Δ*bldD_gh_*Δ*wblA_gh_* was severely impaired in morphological differentiation whereby only the substrate mycelium is formed (Supplementary Figure S6B).

### BldD_gh_ controls the expression of *cdgB_gh_*, *rmdB_gh_* and *wblA_gh_*

Transcriptional analysis reported previously (26,29) suggested that BldD directly interacts with the *cdgB* promoter. To gain further information on the putative interactions of BldD_gh_ with the promoter regions of *cdgB_gh_*, *rmdB_gh_* and *wblA_gh_* we performed EMSA. An *N*-terminally His_6_-tagged BldD_gh_ was incubated with ^33^P-labeled DNA probes corresponding to the promoter of interest. In agreement with an *in silico* identified BldD binding site, BldD_gh_ effectively bound to the *cdgB_gh_* promoter (Figure 3A). Increasing concentrations of BldD_gh_ resulted in a formation of one nucleoprotein complex with the *wblA_gh_* promoter that is consistent with bioinformatics analysis (Figure 3B). Moreover, binding of BldD_gh_ to the *wblA_gh_* promoter was significantly improved in the presence of increasing concentrations of c-di-GMP (Figure 3C). The specificity of this interaction is shown by the ability of unlabeled *wblA_gh_* promoter to compete with the radiolabeled one for BldD_gh_ (Supplementary Figure S9).

EMSA experiments found that the *rmdB_gh_* promoter is not a direct target of BldD_gh_ (Figure 3D). However, analysis of the *rmdB_gh_* coding sequence revealed the presence of one TTA codon, indicating that the translation of the gene is controlled by the tRNA^Leu^_UAA_ molecule encoded by *bldA*. In *S. coelicolor*, mature BldA is only formed during stationary growth and its expression directly controlled by BldD (26). Using a GusA reporter system we first monitored the time dependency of the transcriptional activity of the *rmdB_gh_* promoter in the wild-type strain. GusA activity was detected 13 h after spore inoculation (Figure 3E), then increased to a maximal value after 30 h, which coincides with the stationary phase of cell growth. Subsequently the *rmdB_gh_* promoter activity slowly decreased before reaching a plateau. Transcription of *rmdB_gh_* remained active over the entire time course, suggesting its pivotal role in *S. ghanaensis* life cycle.

We next examined the levels of translation of RmdB_gh_ using a GusA reporter assay. In one construct, encoded by plasmid prmdBtransl, GusA was fused to the *C*-terminus of RmdB_gh_. In a second construct, encoded by the plasmid prmdB-CTGtransl, the TTA codon in *rmdB_gh_* was changed to a synonymic CTG codon. No GusA activity was identified in the first 24 h of growth in wild-type strain carrying prmdBtransl. In contrast, low activity was detectable in the strain carrying prmdB-CTGtransl starting 18 h after inoculation (Figure 3F). Additionally, in *S. ghanaensis* Δ*bldA_gh_* containing prmdBtransl, we observed almost complete cessation of *rmdB_gh_* translation, while in Δ*bldA_gh_* harboring prmdB-CTGtransl translation was unaffected (Figure 3G). These results confirmed the necessity of BldA_gh_ for the efficient translation of the TTA codon.

### c-di-GMP pool affects the expression of cryptic BGCs in *S. ghanaensis*

**Figure 4.**
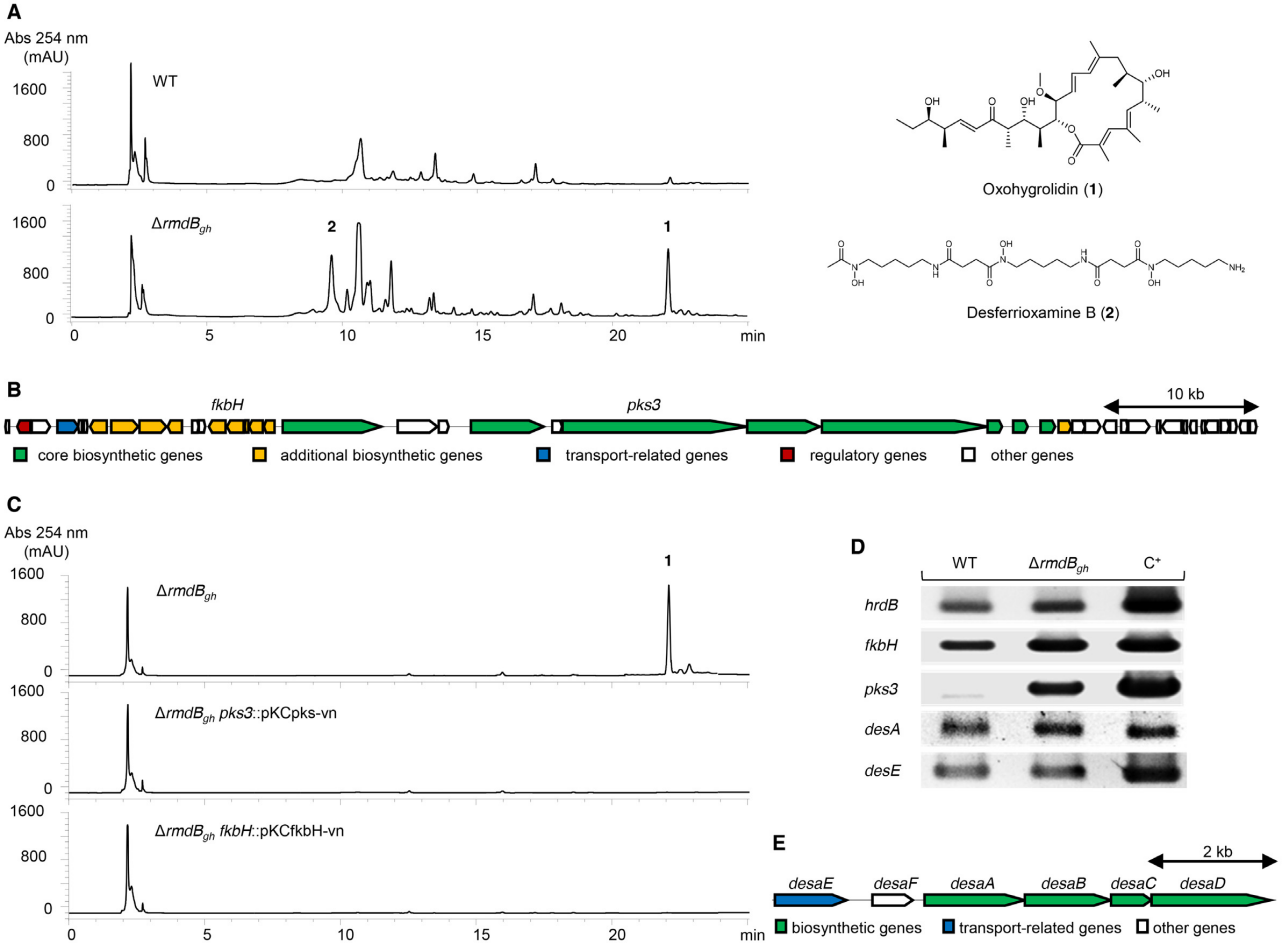
Deletion of *rmdB_gh_* severely affects SMs biosynthesis in *Streptomyces ghanaensis*. (**A**) HPLC chromatograms of butanol extracts derived from *S. ghanaensis* ATCC14672 and *S. ghanaensis* Δ*rmdB_gh_* grown in TSB medium. Several new peaks are observed in the mutant strain. Two most prominent peaks in Δ*rmdB_gh_* were identified as oxohygrolidin (**1**) and desferrioxamine B (**2**). (**B**) Genetic organization of the oxohygrolidin BGC, as identified by AntiSMASH analysis of the *S. ghanaensis* genome. Two structural genes, *fkbH* and *pks3*, inactivated in this work to confirm their relevance to oxohygrolidin biosynthesis are shown. (**C**) HPLC chromatograms of methanol extracts from biomass derived from Δ*rmdB_gh_* and two single-crossover mutants Δ*rmdB_gh_ pks3*::pKCpks-vn and Δ*rmdB_gh_ fkbH*::pKCfkbH-vn. (**D**) sqRT-PCR revealed the strong uplift in expression levels of oxohygrolidin BGC in the *rmdB_gh_*-background compared to the wild-type strain, while the transcription of desferrioxamine B BGC was unaffected. As a template for sqRT-PCR, chromosomal DNA (C^+^) and cDNA obtained from strains grown in TSB for 48 h were used. Total RNA samples were isolated from three independent biological replicates. (**E**) Genetic organization of the desferrioxamine B BGC in *S. ghanaensis* as identified by AntiSMASH analysis.

Although the genome of *S. ghanaensis* contains several BGCs (Supplementary Table S4), the strain is known for its ability to synthesize MmA. Therefore, to explore whether mutations in genes for c-di-GMP turnover have an impact on SM biosynthesis, we investigated extracts of the wild-type strain and the mutant Δ*rmdB_gh_* by HPLC-MS. Interestingly, several peaks were detected in the extract of the Δ*rmdB_gh_* strain which are not present in the extract of the parental strain (Figure 4A). To identify two of the most prominent peaks, we performed large scale fermentation by cultivating Δ*rmdB_gh_* in 4 l of tryptic soy broth. The structure of the first compound was determined by mass spectrometry and NMR spectroscopy to be a macrolide antibiotic oxohygrolidin (**1**) (40) (Figure 4A and Supplementary Note 2). To allocate its BGC in the *S. ghanaensis* chromosome, we employed antiSMASH (38). Among 29 identified putative BGCs (Supplementary Table S4), we focused on a type I PKS encoded by an 86 kb gene cluster that has a high gene synteny and 88% similarity to the bafilomycin BGC (Figure 4B), a compound chemically closely related to oxohygrolidin (40). Inactivation of *pks3* and *fkbH* genes from determined oxohygrolidin BGC, encoding a PKS module and a protein proposed to be responsible for the biosynthesis of unusual PKS extender units, respectively, completely abolished antibiotic production in *S. ghanaensis* Δ*rmdB_gh_* (Figure 4C). Next, the transcriptional profiles of both genes were examined in wild-type and Δ*rmdB_gh_* mutant. Deletion of *rmdB_gh_* elevated *fkbH* transcription, while *pks3* was poorly expressed in the wild-type strain in contrast to the mutant (Figure 4D).

The second compound had a maximum absorbance at 211 nm and a molecular ion of *m/z* 559.3463 [M-H]^–^ (Supplementary Figure S10A). The predicted molecular formula is C_25_H_48_N_6_O_8_, which corresponds to the siderophore desferrioxamine B, a metal-chelating metabolite commonly produced by actinomycetes in response to iron starvation (39). The identity of the peak corresponding to desferrioxamine B was confirmed by MS/MS analysis (Supplementary Figure S10B). The desferrioxamine B gene clusters are ubiquitous in actinobacterial genomes and antiSMASH analysis revealed a putative BGC in *S. ghanaensis* that shows synteny and high percentage of similarity to known desferrioxamine BGCs from streptomycetes (46,47) (Figure 4E and Supplementary Table S4). We assessed the transcription of genes associated with desferrioxamine B metabolism both in wild-type and Δ*rmdB_gh_* mutant. Interestingly, the expression levels of *desaA* and *desaE*, encoding proteins involved in desferrioxamine B biosynthesis and uptake, respectively, were not significantly altered (Figure 4D). Therefore, the underlying cause of the strongly uplifted synthesis of siderophores produced by Δ*rmdB_gh_* remains obscure. Analogous to the study reported by Lambert *et al.* (48), a possible explanation for the high amounts of desferrioxamine B could be reasoned by an impaired expression of genes involved in its export or by post-transcriptional control.

### c-di-GMP-mediated regulation is omnipresent in streptomycetes

**Figure 5.**
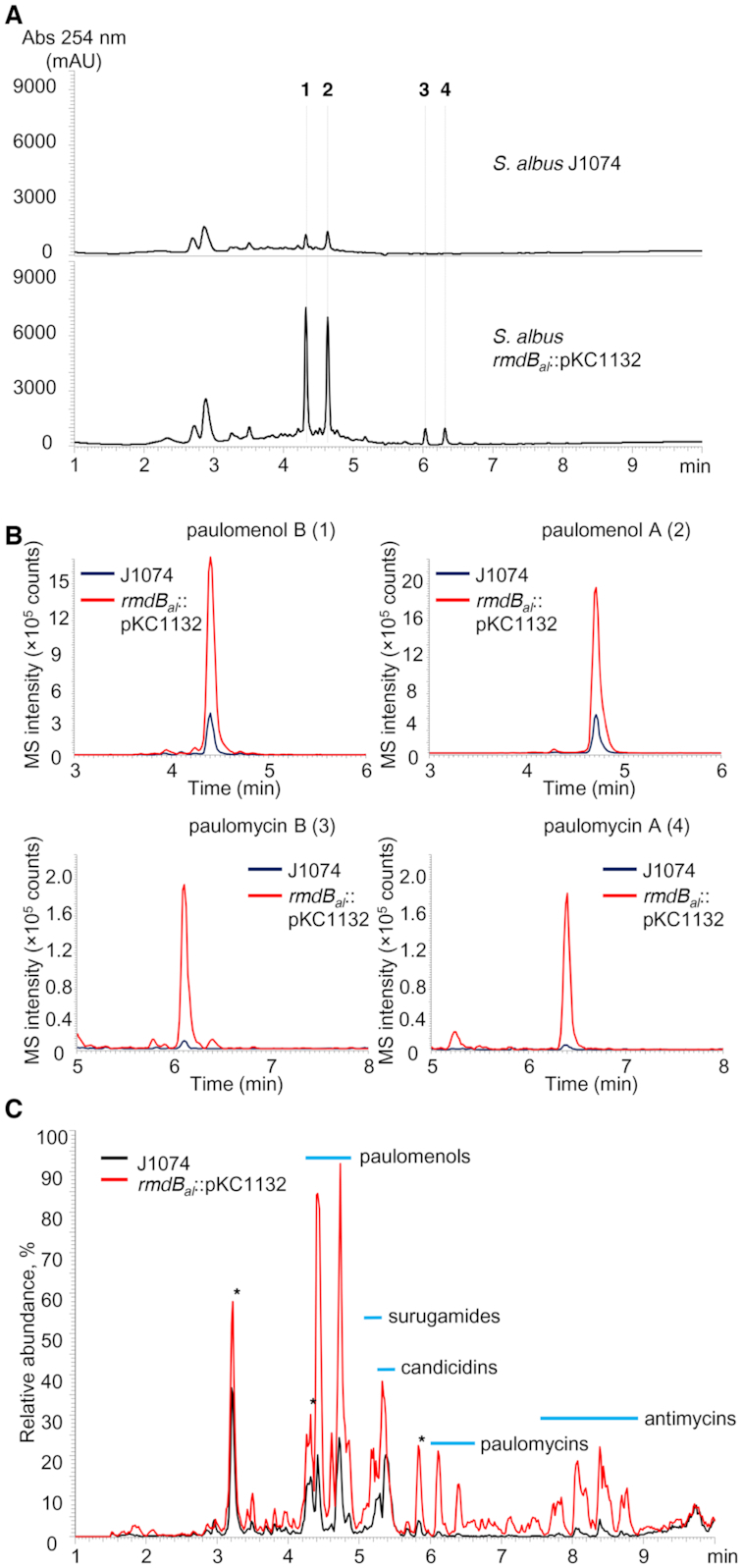
Inactivation of *rmdB_al_* stimulates the production of SMs by *Streptomyces albus*. The images in this figure represent a typical result of five independent HPLC-MS experiments. (**A**) UHPLC chromatograms of ethyl acetate extracts from *S. albus* J1074 and *S. albus rmdB_al_*::pKC1132 grown in R5A medium. The peaks numbering corresponds to paulomenol B (1), paulomenol A (2), paulomycin B (3) and paulomycin A (4). (**B**) Strong induction of SMs biosynthesis in *S. albus rmdB_al_*::pKC1132. Shown are MS extracted ion chromatograms for 1, 2, 3 and 4 from *S. albus* J1074 (dark blue) and *S. albus rmdB_al_*::pKC1132 (red). (**C**) Overlaid UHPLC-MS traces of ethyl acetate extracts derived from *S. albus* strains grown in R5A. Thick blue lines correspond to retention time ranges of detected groups of SMs. Unidentified compounds are marked with asterisks.

C-di-GMP metabolizing ortholog genes can be found in nearly all streptomycetes including the model strain *S. albus* J1074. To investigate whether RmdB also influences SM biosynthesis in this strain, we constructed a mutant of *S. albus* in which the coding sequence of *XNR_1338* (*rmdB_al_*), an ortholog of *rmdB_gh_*, was interrupted by a single-crossover insertion of the plasmid pKCXNR_1338vn. Similar to *S. ghanaensis* Δ*rmdB_gh_*, mutation of *rmdB_al_* severely impaired morphogenesis in *S. albus* (Supplementary Figure S11A). Both wild-type and mutant strains were cultivated in a variety of media for 5 days. Extracts from these cultures were analyzed by HPLC-MS. The production of two compounds (peaks 1 and 2 in Figure 5A) was significantly enhanced in the mutant strain compared to wild-type. Additionally, two new peaks appeared (peaks 3 and 4) in the *rmdB_al_* mutant. These four compounds were found to correspond to paulomenol B (peak 1), paulomenol A (peak 2), paulomycin B (peak 3) and paulomycin A (peak 4) based on absorption spectra, masses and MS/MS-fragmentation patterns (47). Peaks 1 and 2 appeared as degradation products of peaks 3 and 4, respectively (Figure 5A) (47).

A detailed HPLC-MS comparison of the extracts derived from *S. albus* and the *rmdB_al_* mutant revealed a total increase in SMs production in the latter (Supplementary Figure S11B). Production of paulomenol B and A were 6- and 4-fold higher in the mutant compared to the wild-type strain, while production of paulomycin B and A was increased 14- and 17-fold, respectively (Figure 5B). In addition, we observed that candicidins and antimycins were also produced in higher amounts. Interestingly, we detected a substantial increase in the production of surugamides, previously described as cryptic metabolites (49), in the *rmdB_al_* mutant (Figure 5C).

## DISCUSSION

The spread of multidrug resistance among bacterial pathogens is a rising global problem. The appearance of new antibiotics in clinical applications has been limited over the last 35 years (50) mainly because the established techniques used for discovering new antibiotics tend to rediscover known compounds. However, recent investigations have resulted in a plethora of techniques which allow the activation of ‘cryptic’ BGCs (6–10). Generally, some of the new techniques rely on applying of chemical elicitors to bacterial cell cultures, or on co-cultivation with other organisms in order to mimic naturally occurring multispecies interactions in the laboratory conditions with a subsequent thorough comparison of changes in metabolome profiles (49,51–54). However, the activation of BGC requires the presence of inducer and is temporally limited (55). While these strategies are useful for the analytical screening projects, they are less useful for large-scale production. Recent progress in nucleic acid sequencing has led to advances in our understanding of regulatory cascades governing SMs production. This has enabled a new strategy where global pleiotropic regulators are manipulated in order to activate the expression of BGCs (56–60). In this study we applied a unique strategy based on the manipulation of the levels of a ubiquitous bacterial secondary nucleotide messenger c-di-GMP in order to activate the expression of cryptic BGCs in streptomycetes.

Over 30 years have passed since c-di-GMP was first identified. Over this period the role of c-di-GMP has been extended from the allosteric activator of cellulose biosynthesis in *Gluconacetobacter xylinus* (61) to one of the most crucial secondary messengers in bacteria. It has been shown to control numerous processes that allow the bacterium to adapt to rapid environmental or intracellular changes (25,62). The function of c-di-GMP in multicellular streptomycetes remained unclear until 2010 when it was firstly reported to play a role in morphological differentiation (26). A few years later the detailed mechanism controlling morphological progression in *S. venezuelae* was elucidated, where c-di-GMP is required to mediate dimerization of the key pleiotropic regulator BldD in a unique way (27). Both morphogenesis and SM biosynthesis have evolved as a part of a global strategy to survive in unfavorable environmental conditions. Despite the interconnection of these processes, the influence of c-di-GMP on antibiotic production remained obscure.

**Figure 6.**
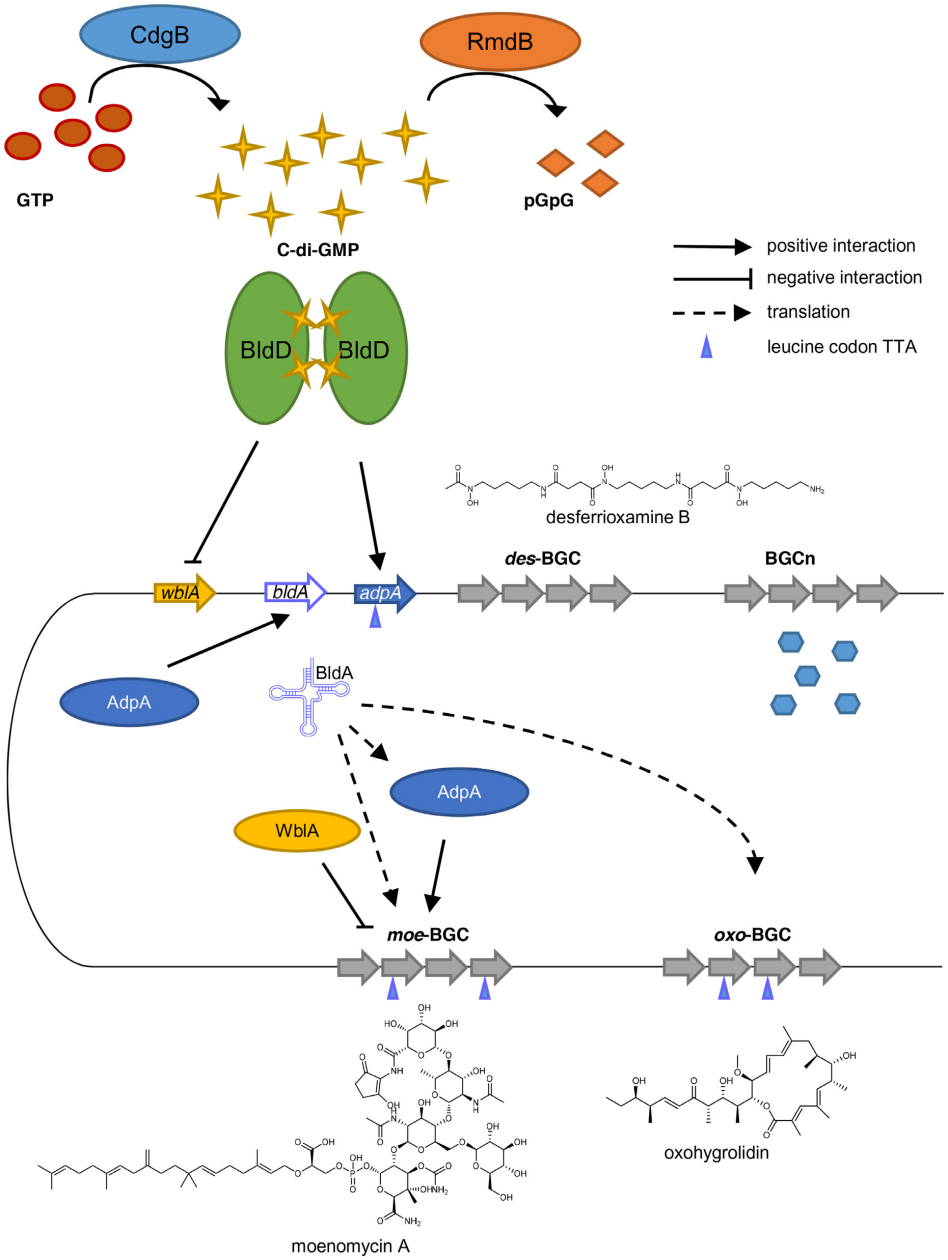
A model of the c-di-GMP mediated regulatory pathway that governs SMs biosynthesis in *Streptomycesghanaensis*.

BldD is one of the most highly conserved regulators in actinomycetes (26,45). In *S. coelicolor* the BldD regulon includes 167 genes (26), while in *S. erythraea* and *Streptomyces roseosporus*, BldD has been shown to control the promoters within the erythromycin and daptomycin BGCs, respectively (63,64). In this study, we demonstrate the pivotal role of c-di-GMP in controlling BGC expression, thereby expanding the c-di-GMP-mediated regulatory network to SM biosynthesis in streptomycetes. The intracellular c-di-GMP level, maintained by the activities of DGCs and PDEs, reflects the physiological state of the bacterium and accordingly regulates morphological differentiation and SM biosynthesis. Crucially, we show that expression of the CSR free MmA gene cluster is governed by c-di-GMP. Deletion of *bldD_gh_* not only reduced MmA production to near zero titers, but also repressed the production of other SMs. We show that deletion of the gene encoding the DGC CdgB_gh_ reduces c-di-GMP levels, which likely promotes the BldD_gh_ dimer to dissociate and, thus, release BldD_gh_ from its target promoters. A drop of cytoplasmic c-di-GMP levels strongly reduces MmA production and also favors morphogenesis. Conversely, elimination of the PDE RmdB_gh_ increases c-di-GMP levels, thereby stimulating SM formation and blocking morphogenesis. The described regulatory network is summarized in Figure 6.

We have also established a key relationship between BldD_gh_ and the regulatory gene *wblA_gh_*. WblA_gh_ belongs to the WhiB family of proteins generally involved in the late steps of morphological differentiation such as sporogenesis (65). In addition, several studies, including that for MmA biosynthesis (24), reported *wblA* orthologs as negative regulators of antibiotic production. Deletion of *wblA* not only strongly increased production of SMs but could also alter the expression of cryptic BGCs (66–69). In this study, we show that deletion of *bldD_gh_* leads to an increase in expression of *wblA_gh_*, which stems from binding of BldD_gh_ to the *wblA_gh_* promoter. Notably, while the *S. ghanaensis* Δ*bldD_gh_* is deficient in MmA production, subsequent deletion of *wblA*_gh_ partially restored MmA biosynthesis. In analogy to *S. ghanaensis*, deletion of *wblA* in *S. roseosporus* doubled the production of daptomycin (69), while the inactivation of *bldD* notably reduced its biosynthesis (64). We propose that the nonproducing phenotypes of *bldD* mutants described in the literature (16,63,70) are partially mediated by high expression of *wblA*, which leads to the repression of antibiotic synthesis.

It is unlikely that only differences in expression levels of *wblA* are solely responsible for the observed phenotypic changes in mutants for c-di-GMP-metabolizing genes. We found that transcription profile of *adpA_gh_*, encoding the positive transcriptional regulator of *moe* genes (23), was greatly influenced in different *S. ghanaensis* mutants. AdpA orthologs are omnipresent in streptomycetes and function as global regulators for secondary metabolism and morphological differentiation (71). In *S. griseus*, the AdpA regulon consists of more than 500 genes (72). In this study, we observed a correlation between intracellular concentration of c-di-GMP and expression level of *adpA_gh_* in various *S. ghanaensis* strains that also influenced MmA production. In contrast to *S. coelicolor*, where transcription of *adpA* is repressed by BldD (26), *adpA_gh_* seems to be positively regulated by BldD_gh_, analogous to the very recent data provided by Yan *et al.* (64). They confirmed that in *S. roseosporus* BldD directly activates transcription of *adpA*, thus expanding the role of BldD in the regulation of SM production and morphological development. Moreover, expression of *bldA_gh_*, encoding the leucine tRNA that reads the UUA codon, was strongly decreased in the Δ*bldD_gh_* mutant. Lower expression of *bldA_gh_* most plausibly originated from the reduced level of AdpA_gh_ since they form a positive regulatory feedback loop where the latter activates the transcription of *bldA_gh_*. At the same time, BldA_gh_ is required for the efficient translation of *adpA_gh_*, which contains a TTA codon that is conserved in all *adpA* sequences (71). In addition to the control by AdpA, in *S. coelicolor* BldD was shown to bind near the *bldA* gene (26) and that may, in turn, also affect the expression of *bldA*. Although the TTA codon is the rarest one in GC-rich streptomycetes genomes, its appearance in regulatory genes and BGCs may confer a regulatory role (73). Several genes within *moe* and oxohygrolidin BGCs include TTA codons subjecting their expression to BldA_gh_-dependent regulation. Based on our findings, we conclude that c-di-GMP-mediated regulatory network coordinating SM production in streptomycetes is immensely intricate and includes several levels of regulation where BldD is located at the top of hierarchy.

Analysis of streptomycetes genomes has revealed the presence of several genes encoding for c-di-GMP turnover enzymes (43). In addition to the catalytic domains DGC and PDE, most of these proteins are also accompanied by additional regions responsible either for spatial localization (transmembrane domains) or for the sensing of specific signals. The presence of additional domains may allow cells to adjust c-di-GMP levels in response to rapidly switching environmental or intracellular signals. Furthermore, the DGC and PDE activities have to be also separated in time-depended manner to permit an efficient and sequential developmental progression. Recently, den Hengst *et al.* found that the expression of three putative DGCs from *S. coelicolor* is under direct control of BldD (26). In this study, we could also show that BldD_gh_ interacts with the *cdgB_gh_* promoter. As in a reciprocal regulatory loop, CdgB_gh_ is proposed to produce c-di-GMP to stimulate the activity of BldD_gh_. When the c-di-GMP pool reaches a certain threshold level, BldD_gh_ represses *cdgB_gh_* transcription. In contrast, BldD_gh_ controls RmdB_gh_ activity not by binding to the *rmdB_gh_* promoter. More likely *rmdB_gh_* expression is indirectly mediated by BldD_gh_ through the regulation of the *bldA_g__h_* encoded tRNA^Leu^_UAA_ molecule. The expression of *rmdB_gh_*, which contains a TTA codon in a position that precedes the EAL domain, is subjected to the translational control from BldA_gh_. The mature BldA_gh_ tRNA appears during the stationary growth phase, thus limiting the translation of the UUA-containing *rmdB_gh_* mRNA to avoid premature PDE activity. Hence, BldD_gh_ is capable to carry out the fine-tuned control of c-di-GMP levels by regulating the expression of both *cdgB_gh_* and *rmdB_gh_*.

Evolutionary analysis suggests that c-di-GMP signaling is an ancient regulatory pathway in prokaryotes and was especially common for early-diverging branches of bacteria (74). The presence of c-di-GMP-metabolizing enzymes in numerous representatives of actinomycetes (43) suggests that the regulatory hierarchy governed by c-di-GMP is widespread. Therefore, manipulation of c-di-GMP levels may be exploited to activate cryptic BGCs in streptomycetes. In this study, deletion of the active PDE RmdB_gh_ in *S. ghanaensis* led to a massive increase in SM production. This includes the macrolide antibiotic oxohygrolidin and the clinically important desferrioxamine B. We also show that this approach is an effective tool to activate the expression of silent BGCs in other streptomycetes, where inactivation of *rmdB_al_* in *S. albus* greatly improved the production yields of a number of SMs. Until now, random mutagenesis and screening were the primary methods of improving yields for compounds encoded by CSR free gene clusters. We propose that manipulation of genes for c-di-GMP turnover is a more efficient way to improve the production titers of such gene clusters.

## Supplementary Material

gkz1220_Supplemental_File
