# Supplementary material for: Secondary nucleotide messenger c-di-GMP exerts a global control on natural product biosynthesis in streptomycetes
Source: Nucleic Acids Res. 2020 Jan 20;48(3):1583–98. doi: 10.1093/nar/gkz1220 (PMC7026642; doi:10.1093/nar/gkz1220)
Supplement: gkz1220_Supplemental_File [file gkz1220_supplemental_file.pdf]

Supplementary Data

Secondary nucleotide messenger c-di-GMP exerts a global control on natural product biosynthesis in streptomycetes

Roman Makitrynskyy[1], Olga Tsypik[1], Desirèe Nuzzo[1], Thomas Paululat[2], David L. Zechel[3] and Andreas Bechthold[1*]

[1]Pharmaceutical Biology and Biotechnology, Institute of Pharmaceutical Sciences, Albert-Ludwigs University, Freiburg, Germany

[2]Organic Chemistry, University of Siegen, Siegen, Germany

[3]Department of Chemistry, Queen's University, Kingston, Ontario, Canada

[*]Correspondence to Andreas Bechthold

SUPPLEMENTARY TABLES

Supplementary Table S1. Strains and plasmids used in this study

Strain / Plasmid	Characteristics	Source / Reference
Strains		
XL1Blue	General cloning host	Agilent
ET12567 (pUZ8002)	Host used for *E. coli-Streptomyces* intergeneric conjugation, methylation deficient	1
BL21 (DE3) Star^{TM}	Host for protein production	Thermo Fisher Scientific
BW25113 (pIJ790)	Host for recombineering experiments	1
S. ghanaensis ATCC14672	Wild type (WT) moenomycin producer	ATCC
S. ghanaensis Δ*bldA_{gh}*	WT derivative, *bldA_{gh}* deletion	2
S. ghanaensis Δ*cdgB_{gh}*	WT derivative, *cdgB_{gh}* deletion	This work
S. ghanaensis Δ*rmdB_{gh}*	WT derivative, *rmdB_{gh}* deletion	This work
S. ghanaensis Δ*bldD_{gh}*	WT derivative, *bldD_{gh}* deletion	This work
S. ghanaensis Δ*wblA_{gh}*	WT derivative, *wblA_{gh}* deletion	This work
S. ghanaensis Δ*bldD_{gh}* Δ*wblA_{gh}*	Δ*bldD_{gh}* derivative, *wblA_{gh}* deletion	This work
S. ghanaensis Δ*rmdB_{gh}* *pks3*:: pKCpks-vn	Δ*rmdB_{gh}* derivative, *pks3* inactivation	This work
S. ghanaensis Δ*rmdB_{gh}* *fkbH*:: pKCfkbH-vn	Δ*rmdB_{gh}* derivative, *fkbH* inactivation	This work
S. albus J1074	Commonly used heterologous host	3
S. albus rmdB_{al}::pKC1132	J1074 derivative, *rmdB_{al}* inactivation	This work
Bacillus cereus ATCC19637	Moenomycin-sensitive test-culture	ATCC
Plasmids		
pET28a(+)	Cloning vector for His-tagged protein production in *E. coli*, kanamycin resistance	Novagen
pET24b	Cloning vector for His-tagged protein production in *E. coli*, kanamycin resistance	Novagen
pET28a-pde	pET28a derived plasmid for production of N-His-tagged PDE-domain of RmdB$_{gh}$ (cytosolic part without DGC-domain)	This work
pET28a-pde-dgc	pET28a derived plasmid for production of N-His-tagged DGC-PDE-domains of RmdB$_{gh}$ (without transmembrane part)	This work
pET28a-dgc	pET28a derived plasmid for production of N-His-tagged DGC-domain of RmdB$_{gh}$ (cytosolic part without PDE-domain)	This work
pET24b-dgc-274	pET24b derived plasmid for production of C-His-tagged DGC-domain of RmdB$_{gh}$ (cytosolic part without PDE-domain)	This work
pET24b-dgc-302	pET24b derived plasmid for production of C-His-tagged DGC-domain of RmdB$_{gh}$ (cytosolic part without PDE-domain)	This work

pBluescript SK(-)	Vector for routine cloning	Addgene
pBlcdgB	pBlueScript carrying *cdgB*$_{gh}$ with 2-kb flanks	This work
pBlrmdB	pBlueScript carrying *rmdB*$_{gh}$ with 3-kb flanks	This work
pBlbldD	pBlueScript carrying *bldD*$_{gh}$ with 3-kb flanks	This work
pBlwblA	pBlueScript carrying *wblA*$_{gh}$ with 3-kb flanks	This work
pBlcdgB::Am	pBlcdgB carrying *cdgB*$_{gh}$ replaced by apramycin cassette	This work
pBlrmdB::Am	pBlrmdB carrying *rmdB*$_{gh}$ replaced by apramycin cassette	This work
pBlbldD::Am	pBlbldD carrying *bldD*$_{gh}$ replaced by apramycin cassette	This work
pBlwblA::Am	pBlwblA carrying *wblA*$_{gh}$ replaced by apramycin cassette	This work
pLERECJ	Carrying apramycin cassette with *loxP*-sites for gene replacement	Prof. Luzhetskyy, Saarland University
pKGLP2	Suicide vector for gene replacement	4
pKGLP2cdgB::Am	*cdgB*$_{gh}$ knockout construct	This work
pKGLP2rmdB::Am	*rmdB*$_{gh}$ knockout construct	This work
pKGLP2bldD::Am	*bldD*$_{gh}$ knockout construct	This work
pKGLP2wblA::Am	*wblA*$_{gh}$ knockout construct	This work
pUWLCre	Carrying *cre* under *ermEp*	5
pKC1132	Suicide vector for gene replacement	6
pKCrmdBal-vn	*rmdB*$_{al}$ inactivation construct	This work
pKCpks-vn	*pks3* inactivation construct	This work
pKCfkbH-vn	*fkbH* inactivation construct	This work
pSET152	φC31-based integrative vector	6
pSETcdgB	pSET152 carrying *cdgB*$_{gh}$	This work
pSETrmdB	pSET152 carrying *rmdB*$_{gh}$	This work
pSETbldD	pSET152 carrying *bldD*$_{gh}$	This work
pKC1139	*Streptomyces* oligocopy vector	7
pKCrmdB	pKC1139 carrying *rmdB*$_{gh}$	This work
pTES	pSET152 carrying *ermEp*	8
pTESacdgB	pTES carrying *cdgB*$_{gh}$	This work
pTESabldD-expI	pTES carrying *bldD*$_{gh}$	This work
pGUS	Promoter probe vector	4
padpAscript	pGUS, *adpA*$_{gh}$*p-gusA* fusion	2
pmoeE5script	pGUS, *moeE5p-gusA* fusion	2
pbldAscript	pGUS, *bldA*$_{gh}$*p-gusA* fusion	2
prmdBscript	pGUS, *rmdA*$_{gh}$*p-gusA* fusion	This work
pGUSHL4aadA	pTES-derivative for translational fusion experiments	4
prmdBtransl	pGUSHL4aadA, *rmdB*$_{gh}$*-gusA* fusion	This work
pSETrmdB-CTG	pSETrmdB, TTA→CTG substitution	This work
prmdB-CTGtransl	pGUSHL4aadA, *rmdB*$_{gh}$(TTA→CTG) -*gusA*	This work

	fusion	
prmdBcontr	pGUSHL4aadA, promoterless *rmdB*$_{gh}$ -*gusA* fusion	This work
prmdB-CTGcontrol	pGUSHL4aadA, promoterless *rmdB*$_{gh}$ (TTA→CTG) -*gusA* fusion	This work
pIJ10257	φBT1-based integrative vector	9
pIJ10350	pIJ10257 carrying *cdgB*$_{sco}$ under *ermEp* control	10

Supplementary Table S2. Oligonucleotides used in this study

Primer name	Sequence	Purpose
cdgB_for	GTGGTCACCCCAGCTCCAG	*cdgB_{gh}* gene deletion
cdgB_rev	CTCCCACGAGCCGCTG	
rmdB_for	aaatctagaGACAACACCTTCAACGACGAC	*rmdB_{gh}* gene deletion
rmdB_rev	aaagaattcCGGTGAAACTTCCCTCTCAG	
bldD-hz-f	AGAAGAGGTTGACCACGGTC	*bldD_{gh}* gene deletion
bldD-hz-r	GTCGAGCTGACCGTCCAG	
wblA_for	aaatctagaCGTTGCCCTGGACCACG	*wblA_{gh}* gene deletion
wblA_rev	aaagatatcCCGAGGAGTACGCCGAGC	
cdgB_kn_for	CTTGATTCACTCCGAGGTCTCGGGGGGAGG GCGAGGATGGATATCTCTAGATACCG	Apramycin cassette for *cdgB_{gh}* replacement
cdgB_kn_rev	CTTGACCTGCGGTTCACCCCGCATGCGACCC GCCGTTCAAACAAAAGCTGGAGCTC	
rmdB_kn_for	CGCGTGGCGTGGGCACCGCCGGCTGTGAGA GGGACGGGAATGGATATCTCTAGATACCG	Apramycin cassette for *rmdB_{gh}* replacement
rmdB_kn_rev	CGCCGACGGCGGACCCCACGGTGTCCGCCT CCGGGGCGTCAAACAAAAGCTGGAGCTC	
bldD_kn_for	AACCCAACCAGCCGCGTCGACACAGTGCCG GGGAGCCATATGGATATCTCTAGATACCG	Apramycin cassette for *bldD_{gh}* replacement
bldD_kn_rev	GCGGTACGTTTCTGCTCGACCCGCGGAAGG CCGTGCGCTCAAACAAAAGCTGGAGCTC	
wblA_kn_for	TTCGTTCAGGGAGCAGCGCAGAACAGGGCC AAGGCGGTGGGATATCTCTAGATACCG	Apramycin cassette for *wblA_{gh}* replacement
wblA_kn_rev	GACCCCCGCGGGTGACCGAGGACCCCTGAG GAACCCTCAAACAAAAGCTGGAGCTC	
xnr_1338_vn1_f	aaatctagaCCTCGACGAGGCCGAACAG	*XNR_1338* disruption
xnr_1338_vn1_r	aaagatatcTCCAGCCCGGCGACGTG	
xnr1338_check	CGACTCCACTCTCTGGATCG	Confirmation of *XNR_1338* disruption
rmdB_EAL_for	GCCGGCACCGGCTACTCCTCC	
pks_vn_for	aaatctagaCTGGTCGCCATCCACCTG	*pks3* disruption
pks_vn_rev	aaagatatcGGAAAGACGAACACCGTCCTG	
fkbH_RT_for	aaatctagaCCGAACGGCTCAACTTCG	*fkbH* disruption
fkbH_RT_rev	aaagatatcGTCGCCAGCAACTTGAGGTG	
cdgB_compl_for	aaatctagaTCCAGGGAGACCGACAG	Δ*cdgB_{gh}* complementation / mutant confirmation
cdgB_compl_rev	aaagaattcTAGGTGCGGATCGAATG	
rmdB_compl_for	aaatctagaTCGAAGAAGACGTCGTTCG	Δ*rmdB_{gh}* complementation / *rmdB_{gh}* overexpression / mutant confirmation
rmdB_exp_for	aaatctagaCTGAGGGTTGTCGGGCATC	
rmdB_exp_rev	aaagaattcGGGTGAGTGTGAGTGGTTTGG	
bldD_compl_for	aaatctagaCGTTCGACGATCTCGTG	Δ*bldD_{gh}* complementation / mutant confirmation
bldD_compl_rev	aaagaattcGGTACGTTTCTGCTCGACC	
cdgB_exp_for	aaaggtaccATTCACTCCGAGGTCTCG	*cdgB_{gh}* overexpression
cdgB_exp_rev	aaagatatcATCCTTCCCTTGACCTGC	
bldD_exp_for1	aaaggtaccGCGTCGACACAGTGCC	*bldD_{gh}* overexpression
bldD_exp_rev	aaagatatcGGTACGTTTCTGCTCGACC	
rmdB_script_rev	aaaggtaccGCCACGCGGCCCGATG	*rmdB_{gh}* promoter

rmdB_compl_for	aaatctagaTCGAAGAAGACGTCGTTCG	amplification / stopcodon-free *rmdB*$_{gh}$ amplification
rmdB_transl_rev	aaagatatcGCCGACCCGCCCCGTG	
rmdB_contr_for	aaatctagaTGGGCACCGCCGGCTG	Promoterless *rmdB*$_{gh}$ amplification
rmdB_CTG_for	CTGCTGCCGGTCGCCGACTC	*rmdB*$_{gh}$ TTA→CTG direct mutagenesis
rmdB_CTG_rev	GACGGCGAACTCGTCG	
wblA_check_rev	aaagaattcCACACGTGACCGCTTCAC	Δ*wblA*$_{gh}$ mutant confirmation/ RT-PCR primers
wblA_RT_for	CCTCGATTCGGGAGAGGAC	
wblA_RT_rev	CGGTCTCCAGCAGCCTG	*wblA*$_{gh}$ RT-PCR primers
desaA_vn_for	CACCCAGTCCAACCTCCAG	*desaA* RT-PCR primers
desA_vn_rev	AGCGTCATCCACAGCTTGAG	
desE_vn_for	CGAGTCCTCGAAGGACAAG	*desaE* RT-PCR primers
desE_vn_rev	GTGCCAGAGACGTAGAAGATC	
moeE5_RT_for	CATCTCGACGGTCTTCCAC	*moeE5* RT-PCR primers
moeE5_RT_rev	ATGGAGACCACTTCGTTGAC	
moeO5_RT_for	GGAAGAGCTTCCTCGAGAC	*moeO5* RT-PCR primers
moeO5_RT_rev	CTGTCGAGGTACTCGGTGA	
moeGT5_RT_for	CTGGACGGACGACGACATC	*moeGT5* RT-PCR primers
moeGT5_RT_rev	CAGAACCAGGTGAAGTGCAG	
adpA_RT_for	GCTCGATCACCTCACCAC	*adpA*$_{gh}$ RT-PCR primers
adpA_RT_rev	AGCGTCTCCACGTCGAAC	
hrdB_gh_for	CGACTACACCAAGGGCTACAA	*hrdB*$_{gh}$ RT-PCR primers
hrdB_gh_rev	TGGTCTTGGACTCGATCTGG	
cdgB_EMSA_for	TCCAGGGAGACCGACAG	*cdgB*$_{gh}$*p* EMSA primers
cdgB_EMSA_rev	CGAGACCTCGGAGTGAATC	
rmdB_EMSA_for	TCGAAGAAGACGTCGTTCG	*rmdB*$_{gh}$*p* EMSA primers
rmdB_EMSA_rev	GCCACGCGGCCCGATG	
wblA_EMSA_f1	GGGCCACGTATCAATACGTCC	*wblA*$_{gh}$*p* EMSA primers
wblA_EMSA_rev	TTCATCCGGATCGGTAGTGC	
BldD-NdeI	AATTAACATATGTCCAGCGAATACGCCAAAC	Production of His-tagged BldD
BldD-XhoI	AAACTCGAGTCAGCTCTCCTCGTGGGAGG	
YdeH-NcoI	AAACCATGGCTATCAAGAAGACAACGGAA	Production of His-tagged YdeH
YdeH-XhoI	TATCTCGAGAACTCGGTTAATCACGTTTT	
EAL_for	AAAAAACATATGGGCCTCACCCTCGTCCTG	Production of His-tagged PDE
EAL_rev	AAACTCGAGTCAGCCGACCCGCCCCG	
GGDEF_for	AAAAAACATATGCAGCTGCGCGACCCGCTG	Production of His-tagged DCG
DGC-274	AATTAACATATGGCCCTGCTCGGCATAGC	
DGC-302	AATTAACATATGGCCCTGGACTCCACCCTG	
GGDEF_rev	AAACTCGAGTCAGTTGGAGTCGCGCTTGGACTC	
GGDEF_for / EAL_rev	See above	Production of His-tagged DGC-PDE

Supplementary Table S3. C-di-GMP turnover proteins encoded by *S. ghanaensis*

Gene	Protein domain architecture	Orthologues
ssfg_00725	PAS-PAC-GGDEF-EAL	*rmdA, sven6830*
ssfg_02181	9 TM-PAS-GGDEF-EAL	*sco5511, sven5187*
ssfg_02196 (*$rmdB_{gh}$*)	6 TM-GGDEF-EAL	*rmdB, sven5165*
ssfg_02343	GGDEF	*sco5345, sven3999*
ssfg_02459	5 TM-HD-GYP	*sven4873*
ssfg_02460	2 TM-HD-GYP *	*sven4872*
ssfg_02707	GAF-GGDEF	*sco4931, sven4602*
ssfg_03956 (*$cdgB_{gh}$*)	GAF-PAS-PAC-GGDEF	*cdgB, sven4034*
ssfg_04551	PAS-GGDEF-EAL	*cdgA, sven2604*

PAS/PAC/GAF-signal domains, GGDEF-cyclic diguanylate cyclase domain, TM-transmembrane domain, EAL/GYP- diguanylate phosphodiesterase domains.

* The entire domain architecture cannot be predicted due to the incomplete genome sequence; partial in the middle of a contig; missing start

Supplementary Table S4. Putative biosynthetic gene clusters encoded by the *S. ghanaensis* genome identified with antiSMASH (ver. 5.0.0beta1-d4ff879)

№	Type	From, bp	To, bp	Most similar known cluster	Similarity
The following regions are from record NZ_DS999641.1					
1	T1PKS	63,385	149,131	Bafilomycin, t1pks	88%
2	T3PKS	177,249	217,834	Alkylresorcinol, t3pks	100%
3	NRPS	262,340	319,857	Stenothricin, NRPS	13%
4	bacteriocin	582,746	590,945	Informatipeptin, lanthipeptide	28%
5	NRPS	803,107	868,453	Laspartomycin, NRPS	20%
6	terpene	987,917	1,012,309	Hopene, terpene	84%
7	phosphoglycolipid	1,397,592	1,425,782	Teichomycin, other	77%
8	siderophore	1,522,750	1,532,083		
9	terpene	1,534,488	1,554,917		
10	ladderane, arylpolyene, NRPS	1,555,405	1,674,468	Skyllamycin, NRPS	48%
11	terpene	1,772,312	1,791,540	Geosmin, terpene	100%
12	bacteriocin	1,811,766	1,822,674		
13	T1PKS	1,827,870	1,875,244	Enduracidin, NRPS	29%
14	siderophore	2,146,205	2,157,801		
15	terpene	2,717,248	2,736,624	Albaflavenone, terpene	100%
16	siderophore	4,982,725	4,994,497	Desferrioxamine, other	66%
17	melanin	5,060,112	5,070,567	Melanin, other	100%
18	lassopeptide	5,557,477	5,579,934		
19	ectoine	6,039,673	6,050,071	Ectoine, other	100%
20	NRPS,T2PKS	6,867,521	6,981,858	Spore pigment, t2pks	66%
21	terpene	7,189,411	7,214,649	Carotenoid, terpene	54%
22	lanthipeptide	7,266,431	7,289,088	SapB, lanthipeptide	75%
23	NRPS,amglyccycl	7,483,579	7,534,897	Guadinomine, nrps-t1pks	7%
24	T3PKS,fused	7,647,895	7,688,893	Pheganomycin, nrps-ripp	47%
25	NRPS-like,furan	7,745,399	7,788,860	Elaiophylin, t1pks	20%
26	bacteriocin	7,882,687	7,894,588		
27	hglE-KS	7,916,828	7,965,229	Esmeraldin, other	8%
The following regions are from record NZ_DS999642.1					
28	other, NRPS, T1PKS, butyrolactone	3,189	102,884	C-1027, polyketide	62%
29	other, T1PKS, NRPS	168,304	231,283	Merochlorin, t3pks-terpene	19%

SUPPLEMENTARY FIGURES

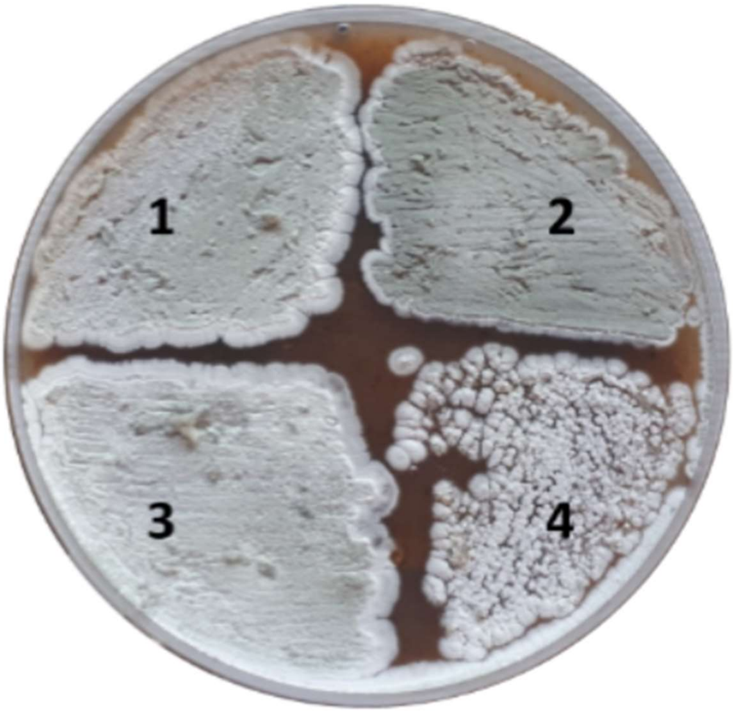

Supplementary figure S1. *S. ghanaensis* Δ$cdgB_{gh}$ (**2**) displays precocious onset into sporogenesis compared to the wild type strain (**1**). Overexpression of $cdgB_{gh}$ (**4**) blocked development at aerial mycelium level compared to a strain bearing an empty vector (**3**). Strains were grown for 4 days on the oatmeal agar medium.

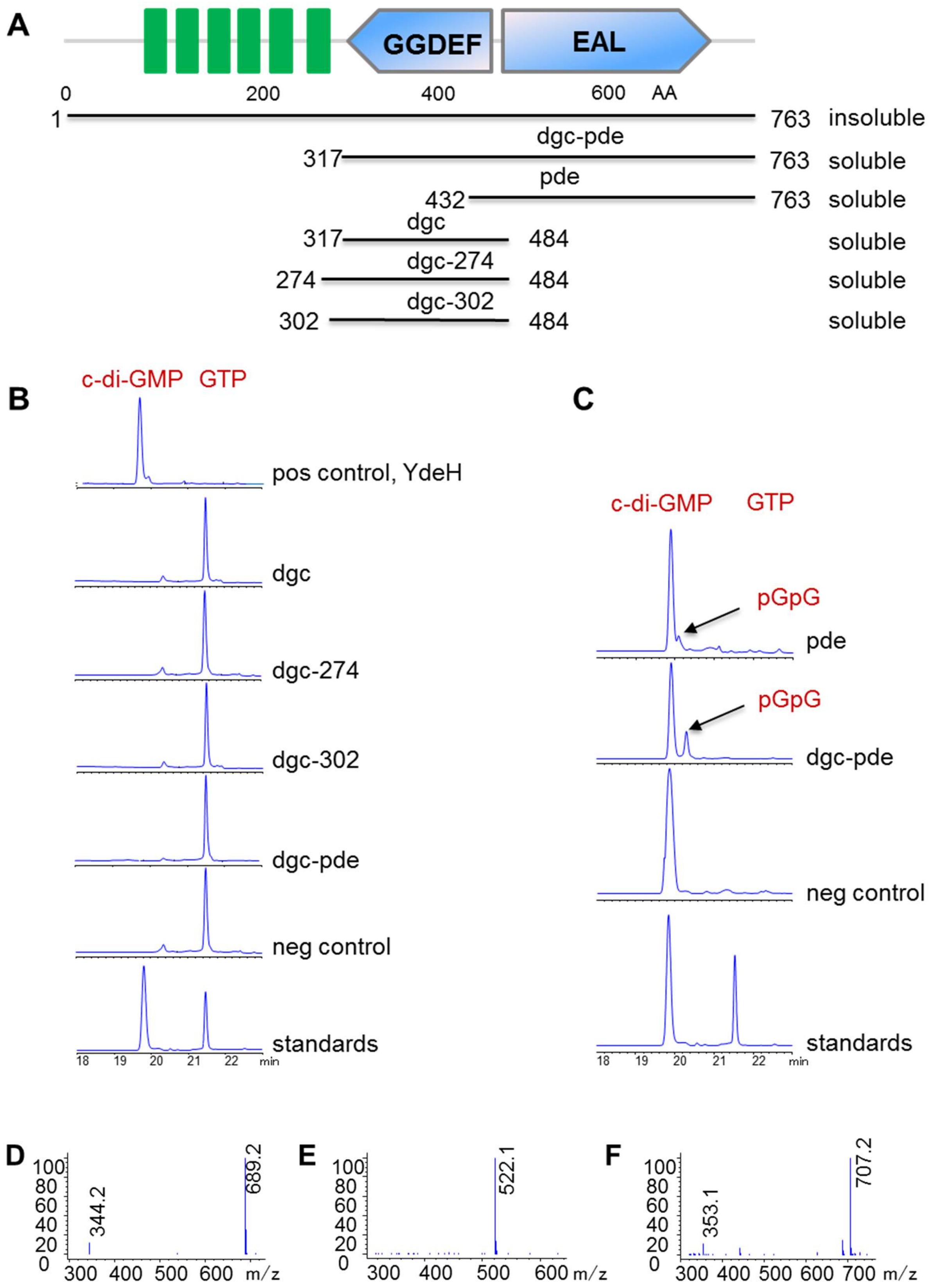

Supplementary figure S2. *In vitro* enzymatic assays with differently truncated versions of $RmdB_{gh}$ revealed only PDE activity. (**A**) General scheme of the differently truncated $RmdB_{gh}$ production. (**B**) HPLC chromatograms of *in vitro* reactions to test DGC activity. (**C**) HPLC chromatograms of *in vitro* reactions to test PDE activity. MS spectra of peaks (negative mode) corresponding to c-di-GMP (**D**), GTP (**E**) and pGpG (**F**).

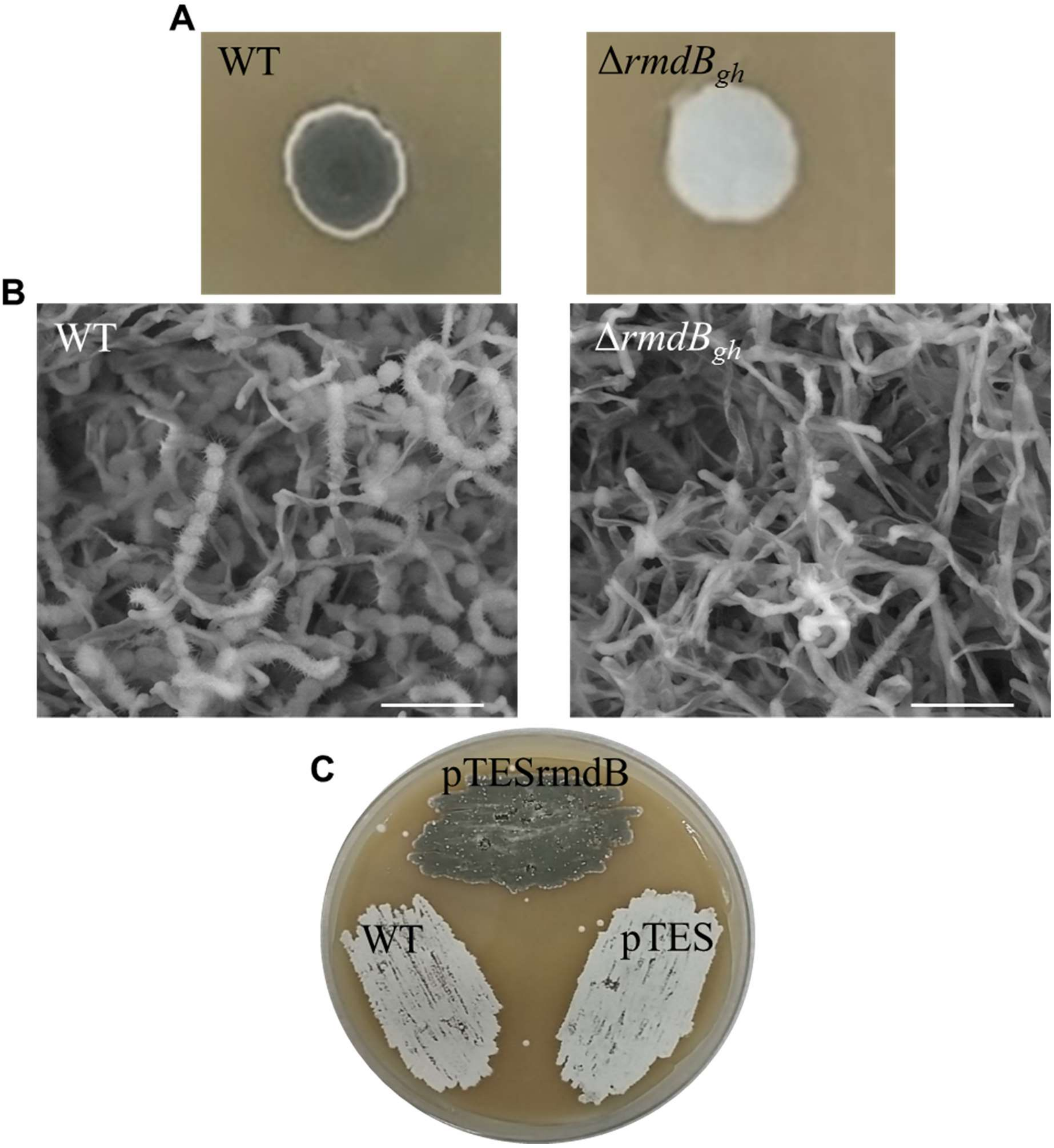

Supplementary figure S3. $RmdB_{gh}$ activity is crucial for normal morphogenesis. Sporulation is impaired in the *S. ghanaensis* $\Delta rmdB_{gh}$ mutant. (**A**) Colonies of *S. ghanaensis* strains were grown on the SFM medium for 5 days. (**B**) Surfaces of the colonies from **a** were used to obtain SEM images. Scale bars are 5 µm. (**C**) Extra copies of $rmdB_{gh}$ enhances sporulation in *S. ghanaensis* grown on SFM for 43 hours.

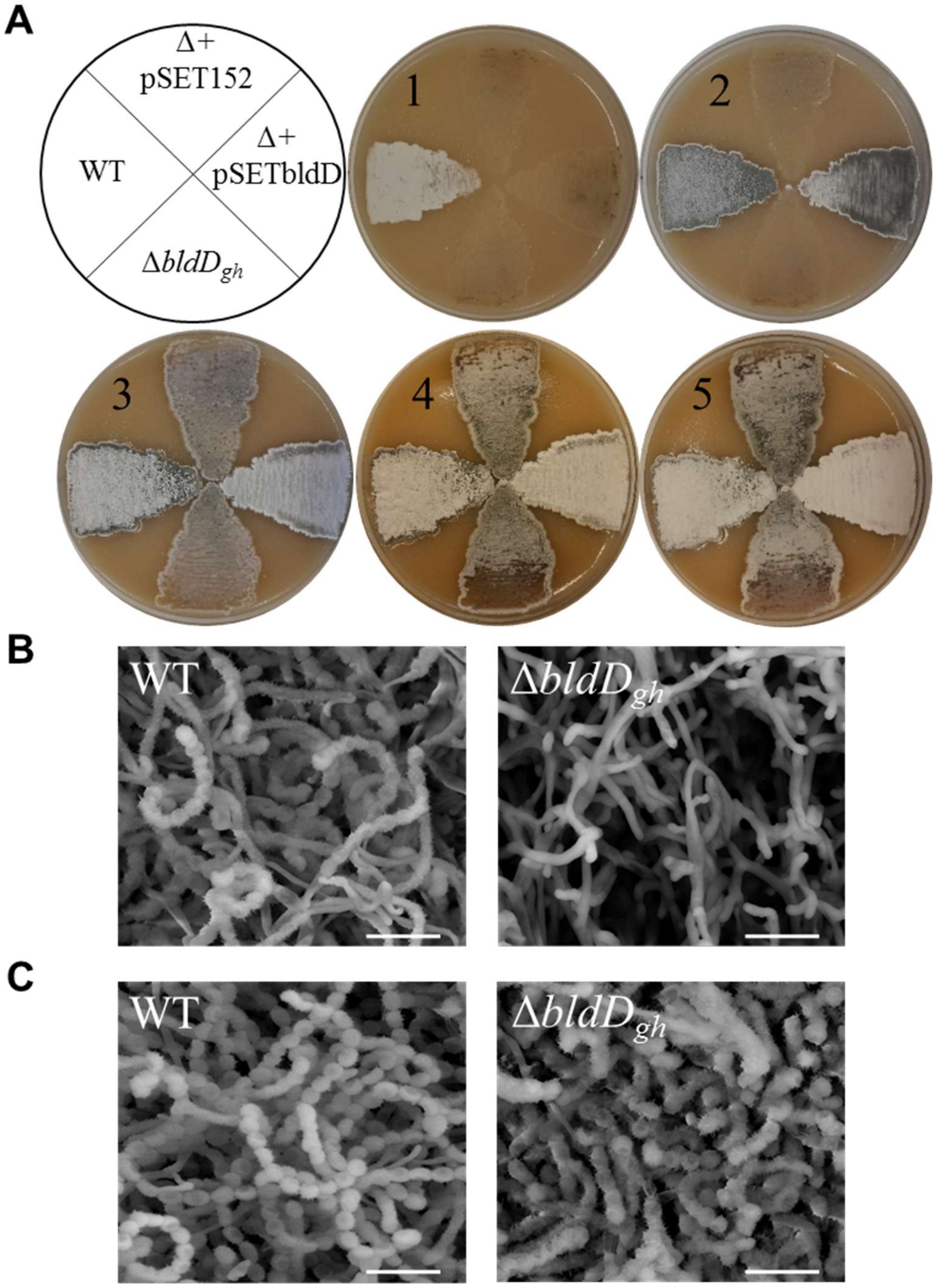

Supplementary figure S4. BldD$_{gh}$ controls the timing of morphological development. (**A**) Lawns of *S. ghanaensis* strains grown over the time course. Strains were cultivated on SFM for 5 days. Pictures were captured each 24 hours. Surfaces of the lawns from **A** were used to obtain SEM images. (**B**) SEM pictures taken on the second day. (**C**) SEM pictures taken on the fifth day. Scale bars are 5 µm (**B** and **C**).

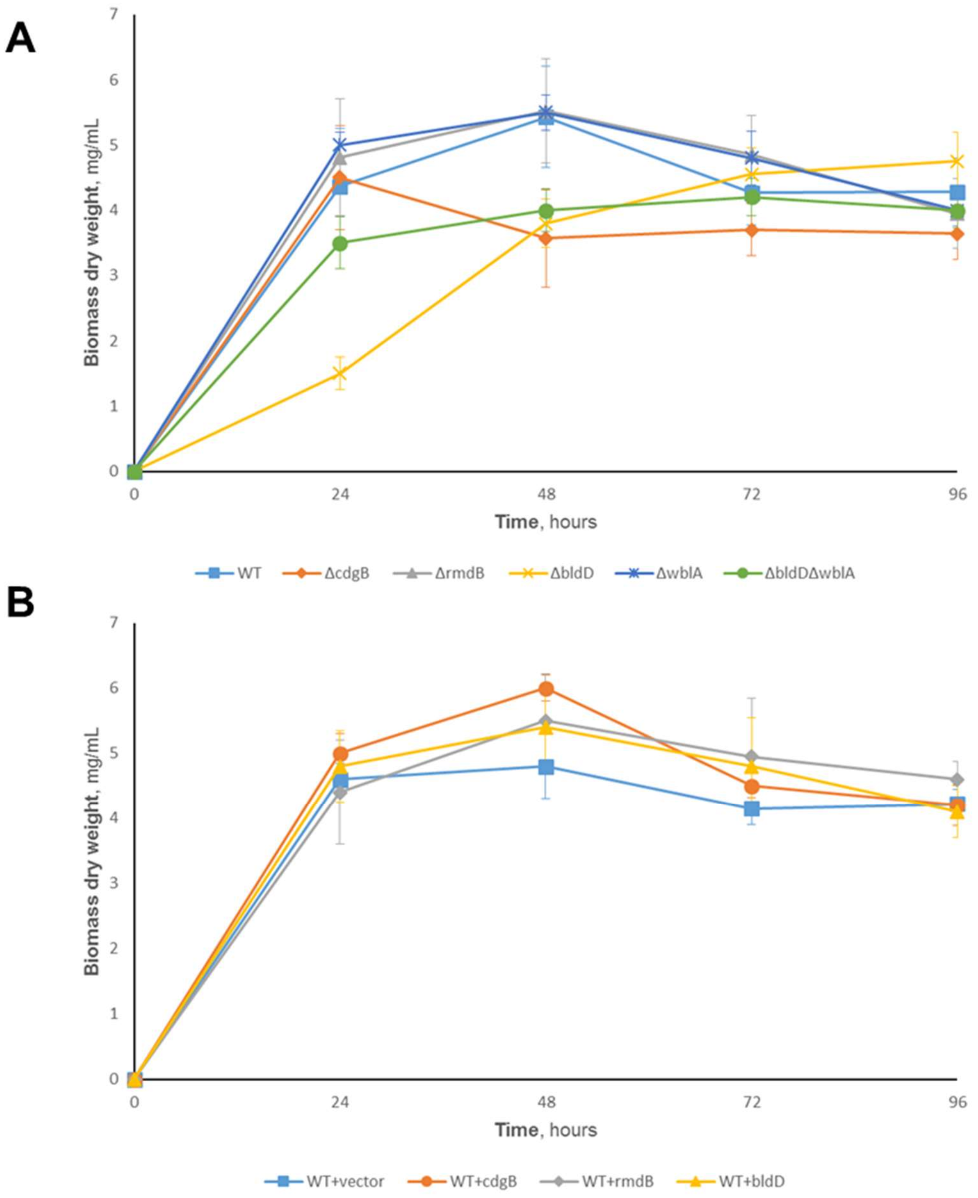

Supplementary figure S5. Growth curves of *S. ghanaensis* strains with gene deletions (**A**) and overexpressions (**B**) studied in this work. The experiment was essentially performed as described before[11]. The experiment was done twice in three replicates each time. Error bars, ±2 SD.

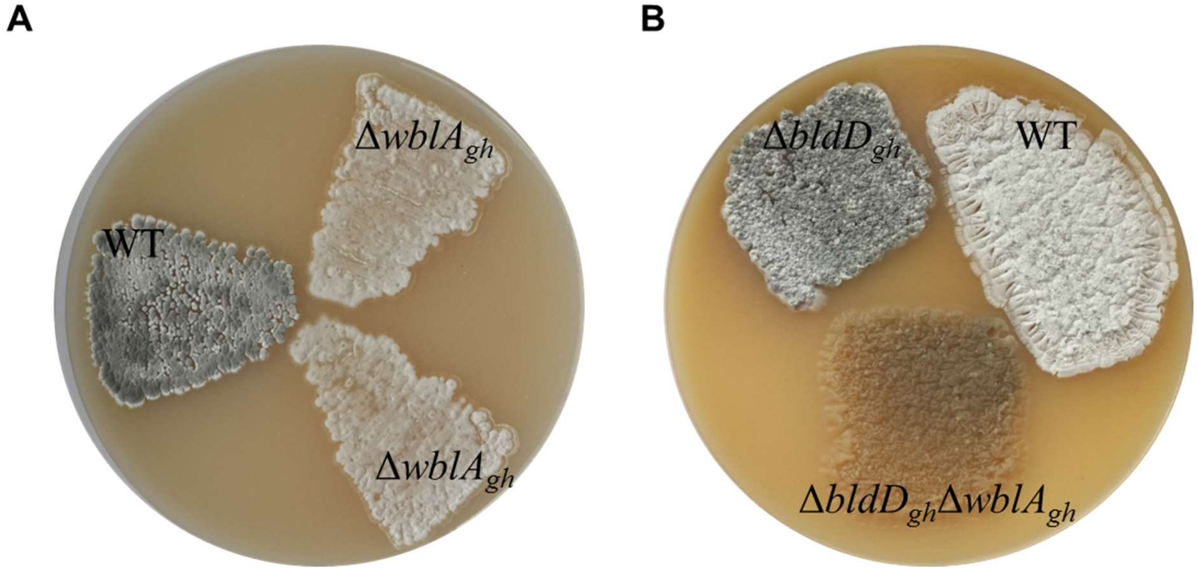

Supplementary figure S6. (**A**) *S. ghanaensis* Δ*wblA*$_{gh}$ mutant displayed "white" phenotype due to inability to form mature spores. Strains were grown on SFM medium for 3 days. (**B**) Deletion of *wblA*$_{gh}$ from the *S. ghanaensis* Δ*bldD*$_{gh}$ chromosome strongly impaired morphological development. Strains were grown on SFM medium for 5 days.

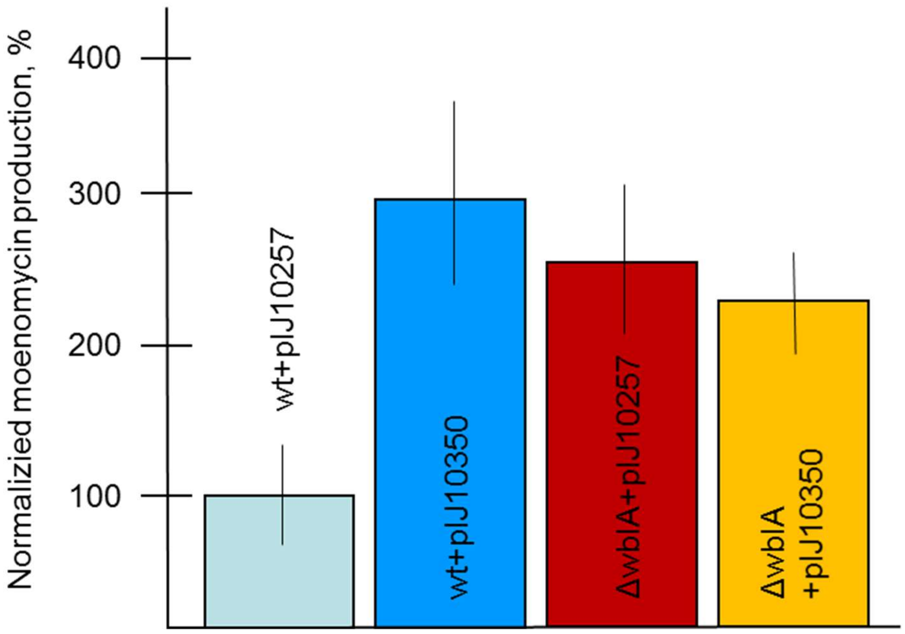

Supplementary figure S7. Levels of moenomycin production by various *S. ghanaensis* strains as determined by HPLC-MS. The mean value of moenomycin mass peak area in *S. ghanaensis* ATCC14672 was taken as 100%. Amounts of moenomycin were normalized to equal amounts of biomass (dry weight) and are the mean value from at least three independent biological replicates. Error bars, ±2 SD.

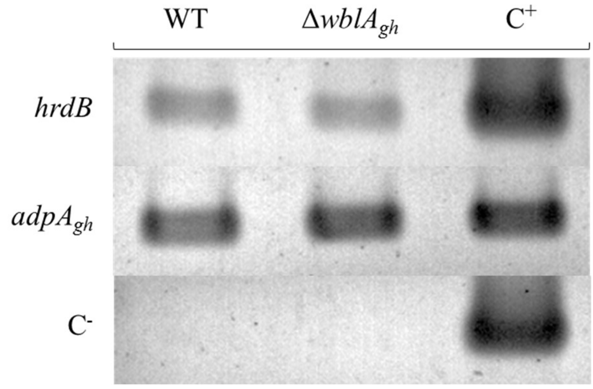

Supplementary figure S8. Deletion of *wblA*$_{gh}$ does not influence expression of *adpA*$_{gh}$. Comparison of Δ*wblA*$_{gh}$ and *S. ghanaensis* ATCC14672 transcriptional profiles. The expression of tested genes was analyzed in 48 h cultures grown in TSB; 200 ng of RNA sample were used per reaction; C^+, positive control (genomic DNA of ATCC14672 strain). Attempts to synthesize *hrdB* from RNA without pretreatment with RT served as negative controls (marked as C^-). Total RNA samples were isolated from three independent biological replicates. The images represent the typical result of three independent RT-PCR experiments.

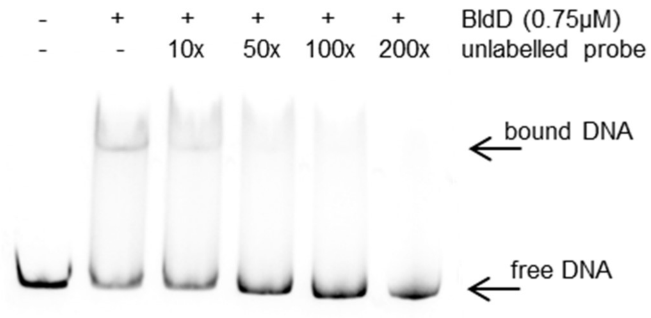

Supplementary figure S9. EMSA competition assay of $BldD_{gh}$ with $wblA_{gh}p$. The reaction was carried out with 0.75 µM of purified $BldD_{gh}$, 1.5 µM c-di-GMP, 20 fmol of ^{33}P-labeled $wblA_{gh}p$ and increasing concentrations of unlabelled probe (10-, 50-, 100- and 200-fold molar excess to labelled probe).

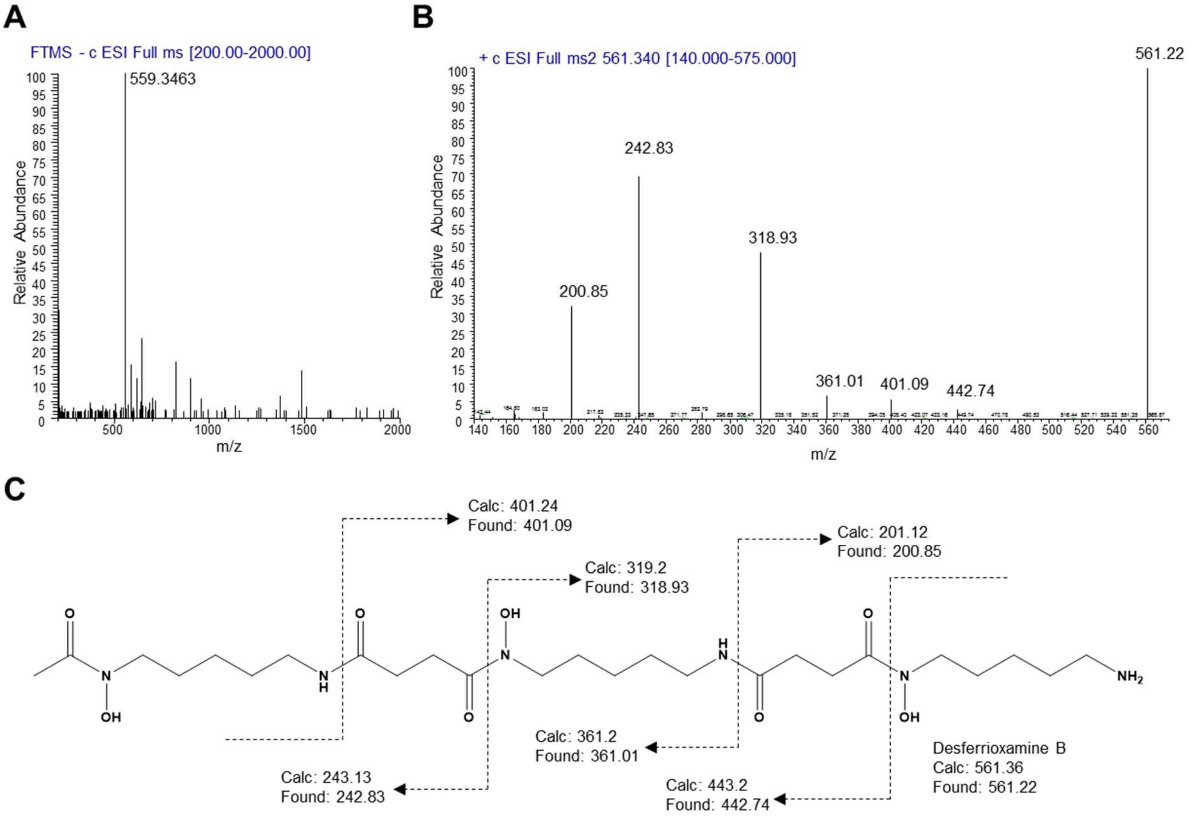

Supplementary figure S10. (A) HRMS spectrum of desferrioxamine B. (**B**) ESI-MS/MS fragmentation pattern of desferrioxamine B. (**C**) Proposed ESI-MS/MS fragmentation of desferrioxamine B.

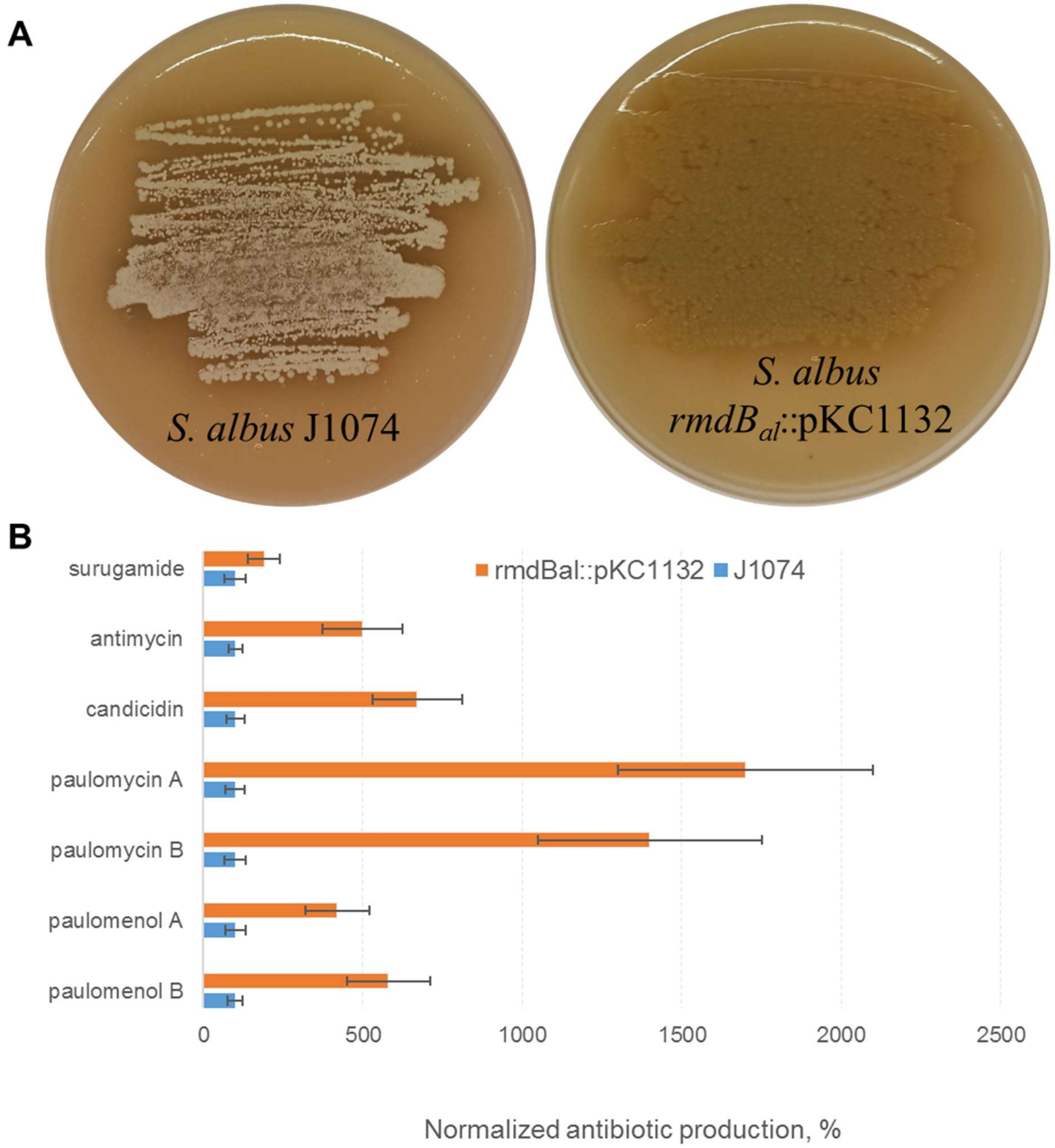

Supplementary figure S11. Inactivation of *rmdB*$_{al}$ in the *S. albus* chromosome strongly affected morphological development and SM production. (**A**) Morphology of strains grown on SFM medium for 4 days. (**B**) Comparison of antibiotic production titers by the *S. albus* strains as determined by HPLC-MS. The mean value of antibiotic mass peak area in *S. albus* J1074 was taken as 100%. Amounts of compounds were normalized to equal amounts of biomass (dry weight) and are mean values from at least three independent biological replicates. Error bars, ±2 SD.

Supplementary Note 1. List of abbreviations and acronyms used in the work.

(sq)RT-PCR	semiquantitative reverse transcription polymerase chain reaction
aac(3)IV	apramycin resistance gene
antiSMASH	"annotation and analysis of secondary metabolite biosynthesis gene clusters" tool
BGC	biosynthetic gene cluster
bld	"bald" genes responsible for aerial mycelium formation
BldDbs	conservative BldD-binding site
cdg	cyclic dimeric GMP genes
cfu	colony-forming unit
Cre	Cre recombinase
CSR	cluster situated regulator
DGC	diguanylate cyclases
EMSA	electrophoretic mobility shift assay
ermEp	strong constitutive promoter of *ermE* gene from the erythromycin BGC
ESI	electrospray ionization
FIMO	"Find Individual Motif Occurences" software
GusA	reporter system based on β-glucuronidase activity
HPLC	High Performance Liquid Chromatography
HRMS	High Resolution Mass Spectrometry
hyg	hygromycin resistance gene
LC–MS	liquid chromatography–mass spectrometry
loxP	recognition site for Cre recombinase
MEME	"Multiple Em for Motif Elicitation" software
MmA	moenomycin A
moe cluster	moenomycin A biosynthetic gene cluster
MS/MS	tandem mass spectrometry
NMR	nuclear magnetic resonance
PDE	phosphodiesterase
pGpG	5'-phosphoguanylyl-(3'→5')-guanosine
PKS	polyketide synthase
PVDF	polyvinylidene difluoride
REDIRECT	gene replacement system, based on λ RED (*gam, bet, exo*) function
rmd	regulator of morphology and development genes
SEM	scanning electron microscopy
SM	secondary metabolite
UHPLC	Ultra-High Performance Liquid Chromatography
whi	"white" genes responsible for sporogenesis

Supplementary Note 2. Chemical characterization data for oxohygrolidin.

NMR-Data for oxohygrolidin in CD_3OD (600/150MHz, CD_3OD, 25°C)

Pos	δ_C	δ_H (*J* Hz)	COSY[a]	HMBC[a]
1	172.4			3-H, 15-H, 26-H_3
2	123.1			3-H, 26-H_3, (27-H_3)
3	147.8	7.25 s	5-H, 26-H_3	5-H, 26-H_3, 27-H_3
4	134.5			3-H, (5-H), (7-H), 6-H, 26-H_3, 27-H_3
5	146.7	5.95 d (8.8)	3-H, 6-H, 27-H_3	3-H, 7-H, 6-H, (26-H_3), 27-H_3, 28-H_3
6	38.7	2.54 ddq (8.8, 1.8, 7.2)	5-H, 7-H, 28-H_3	7-H, 8-H, 28-H_3
7	81.3	3.26 dd (6.4, 2.0)	6-H, 8-H	5-H, 8-H, 9-H_2, 28-H_3, 29-H_3
8	41.1	1.87 m	7-H, 9-H_2, 29-H_3	7-H, 9-H_2, 29-H_3
9	42.6	2.02	11-H, 30-H_3	11-H, 29-H_3, 30-H_3
10	144.3			9-H_2, (11-H), 12-H, (13-H), 29-H_3, 30-H_3
11	125.4	5.76 d (10.8)	12-H, 30-H_3	9-H_2, 12-H, 13-H, 30-H_3
12	134.1	6.55 dd (15.2, 10.8)	11-H, 13-H	11-H, 14-H, 30-H_3
13	126.8	5.13 dd (14.8, 8.4)	12-H	11-H, (12-H), 13-H, 14-H, 30-H_3
14	85.4	3.93 dd (8.4, 7.2)	15-H	9-H_2, 12-H, 13-H, 34-H_3
15	76.86	5.11 dd (6.8, 2.0)	14-H, (16-H)	13-H, 14-H, 17-H, 31-H_3
16	40.6	2.00	(15-H), 17-H, 31-H_3	14-H, 15-H, (17-H), 31-H_3
17	73.8	3.75 dd (8.8, 4.0)	16-H, 18-H	15-H, 16-H, 18-H, 31-H_3, 32-H_3
18	47.6	3.06 dq (4.0, 6.8)	17-H, 32-H_3	16-H, (17-H), 32-H_3
19	205.0			17-H, 18-H, 20-H, 21-H, 32-H_3
20	129.5	6.24 dd (14.9, 0.8)	21-H, (22-H)	(21-H), 22-H
21	151.3	6.82 dd (15.7, 8.0)	20-H, 22-H	20-H, 23-H, 22-H, 33-H_3
22	44.2	2.38 m	21-H, (23-H), 33-H_3	20-H, 21-H, 24-H_b, 33-H_3
23	76.89	3.37 ddd (9.2, 6.0, 3.6)	22-H, 24-H_b	(20-H), 21-H, 22-H, 24-H_b, 25-H_3, 33-H_3
24	28.6	H_a: 1.50 m<br>H_b: 1.32 m	22-H, 14-H_b, 25-H_3,<br>22-H, 14-H_a, 25-H_3	22-H, 25-H_3
25	10.7	0.93 t (7.2)	24-H_a, 24-H_b	(23-H), 24-H_b
26	14.1	2.03 s	3-H	3-H, ^1J
27	15.4	1.96 d (0.8)	5-H	3-H, 5-H, ^1J
28	18.3	1.06 d (7.2)	6-H	6-H, 7-H, 27-H_3, ^1J
29	22.5	0.92 d (6.4)	8-H	7-H, 8-H, 9-H_2
30	19.8	1.85 s	9-H_2, 11-H	9-H_2, 11-H
31	11.3	0.97 d (6.8)	16-H	15-H, 16-H, ^1J
32	9.8	1.10 d (6.8)	18-H	17-H, 18-H, ^1J
33	15.4	1.08 d (6.8)	22-H	21-H, 22-H, ^1J
34	56.0	3.22 s		14-H, ^1J

[a]Weak signals in brackets

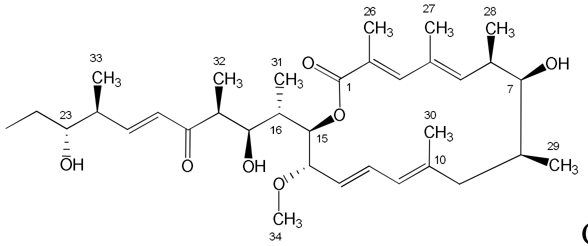

Calc $[M+Na]^+$: 597.3762; found 597.3754

Oxohygrolidin was firstly identified by Kretschmer et al.[12]

HRMS analysis (positive mode) of oxohygrolidin.

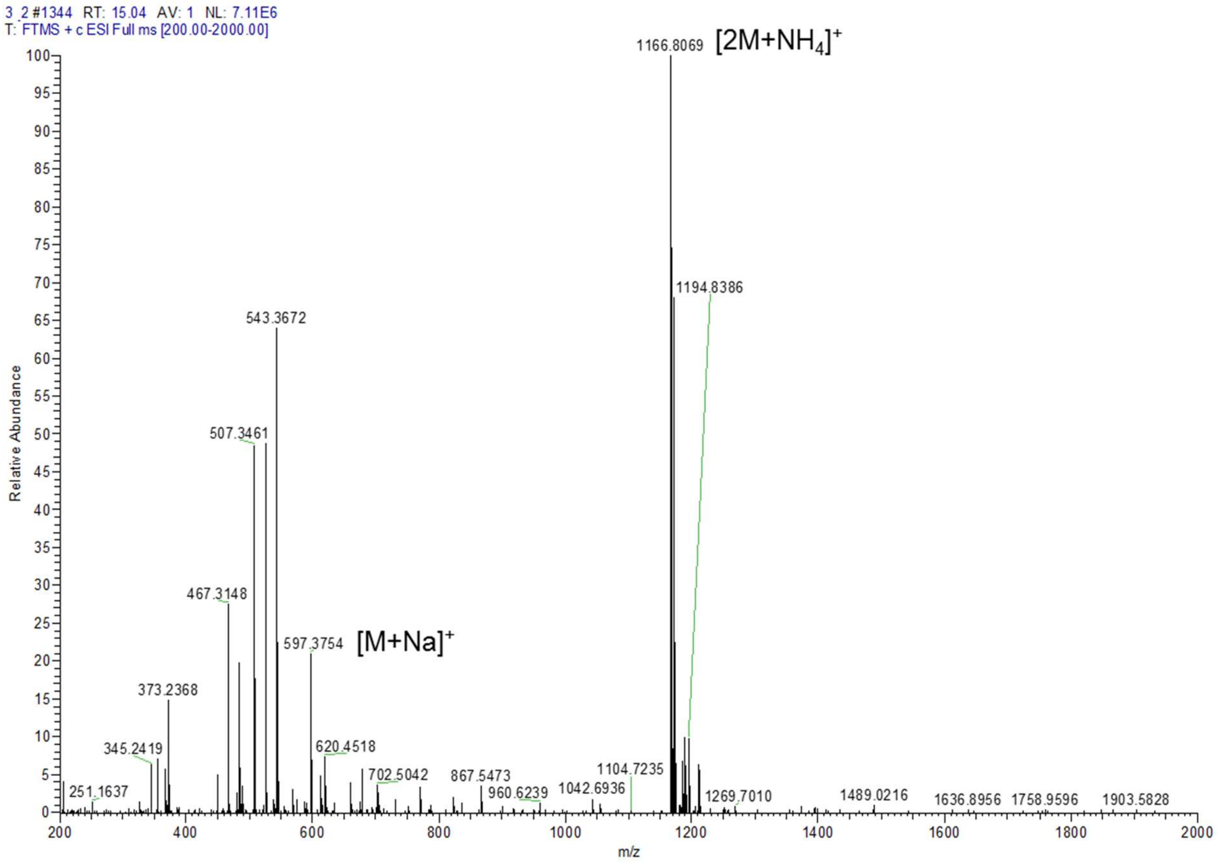

^{1}H NMR spectrum of oxohygrolidin (600 MHz, CD_3OD, 25 °C).

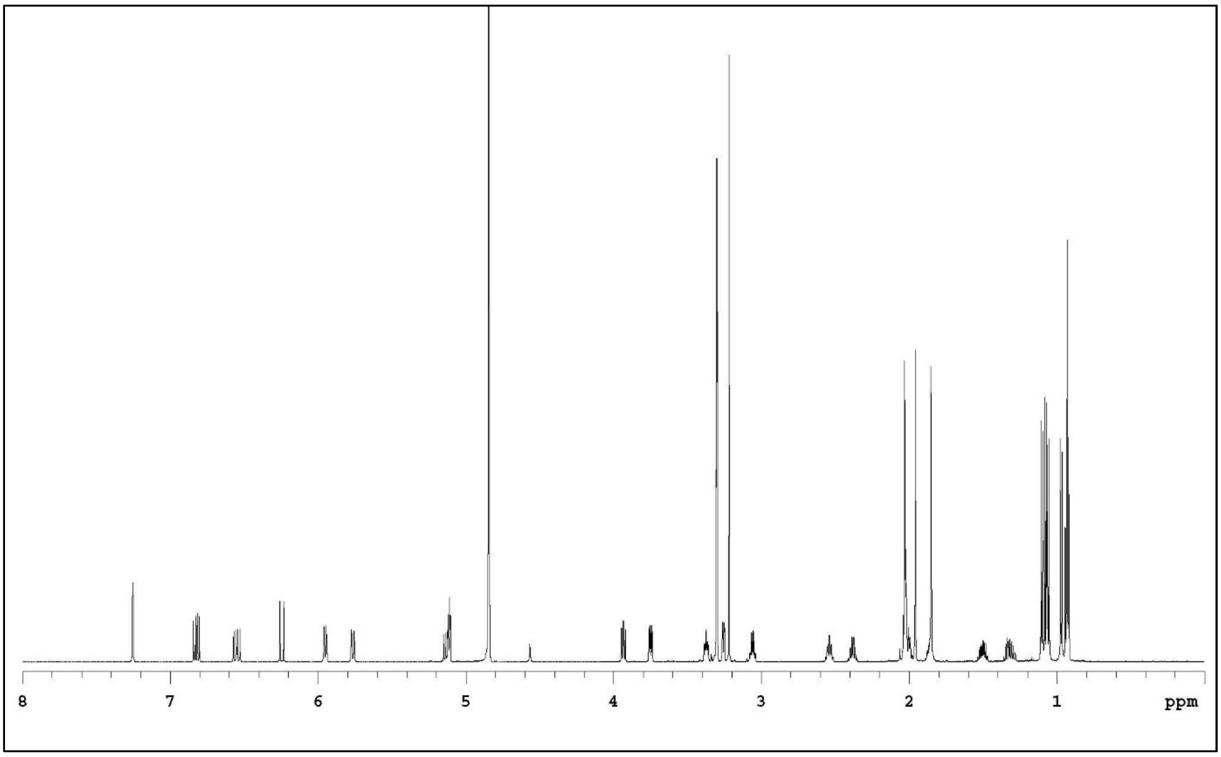

^{13}C NMR spectrum of oxohygrolidin (150 MHz, CD_3OD, 25 °C).

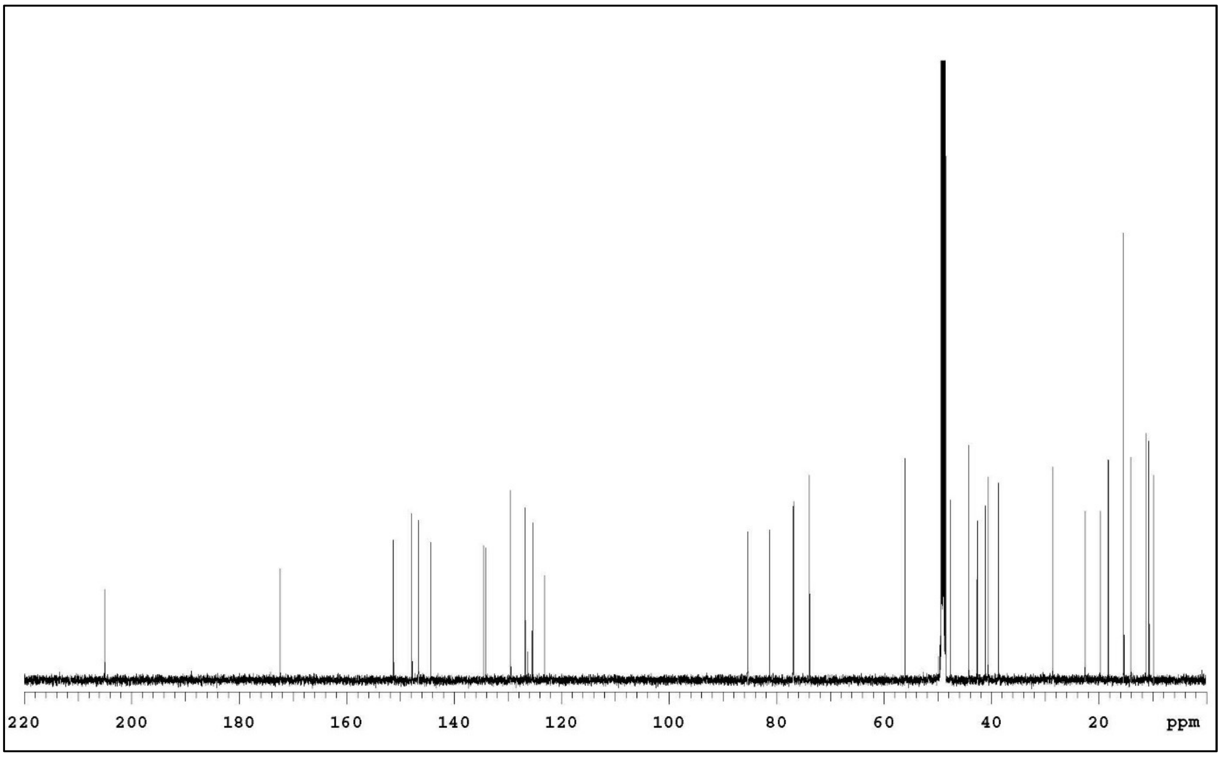

DEPT spectrum of oxohygrolidin (150 MHz, CD_3OD, 25 °C).

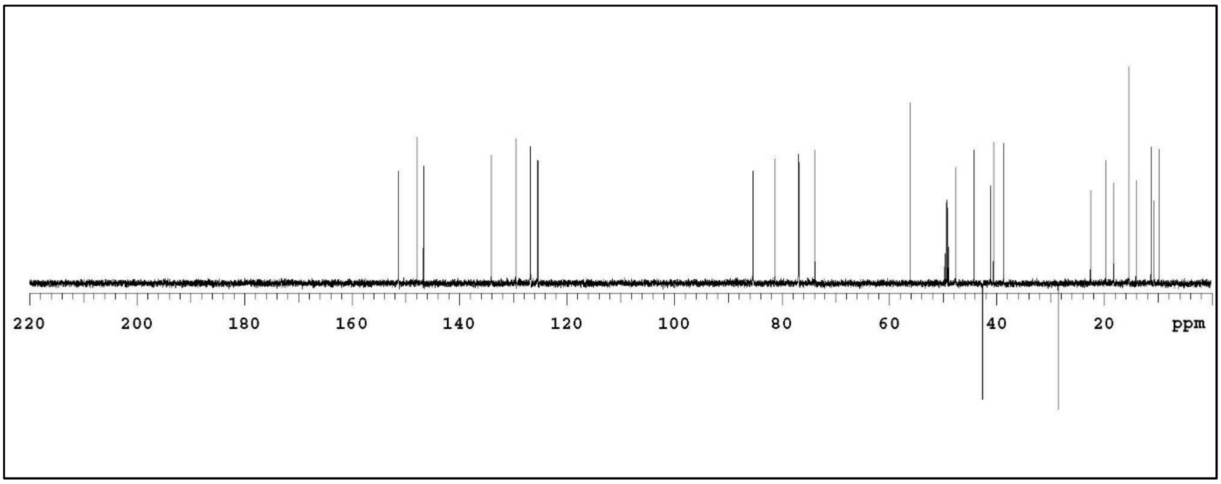

COSY spectrum of oxohygrolidin (600 MHz, CD3OD, 25 °C).

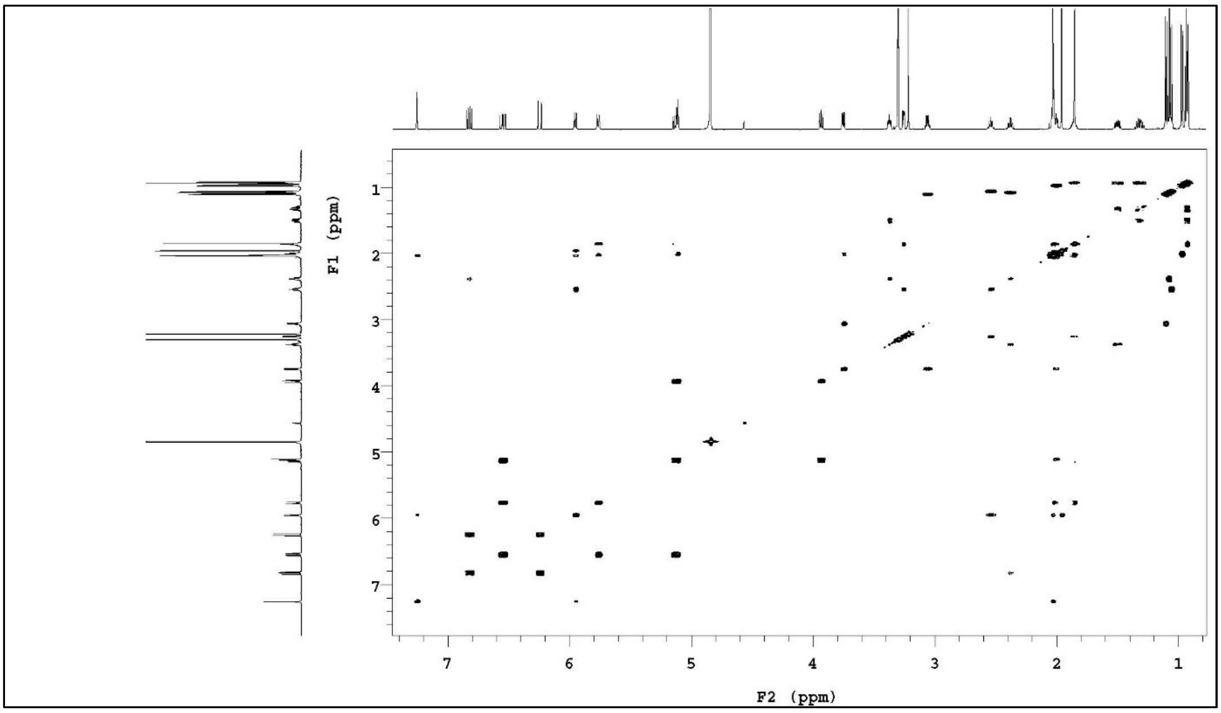

HSQC spectrum of oxohygrolidin (600 MHz, CD_3OD, 25 °C).

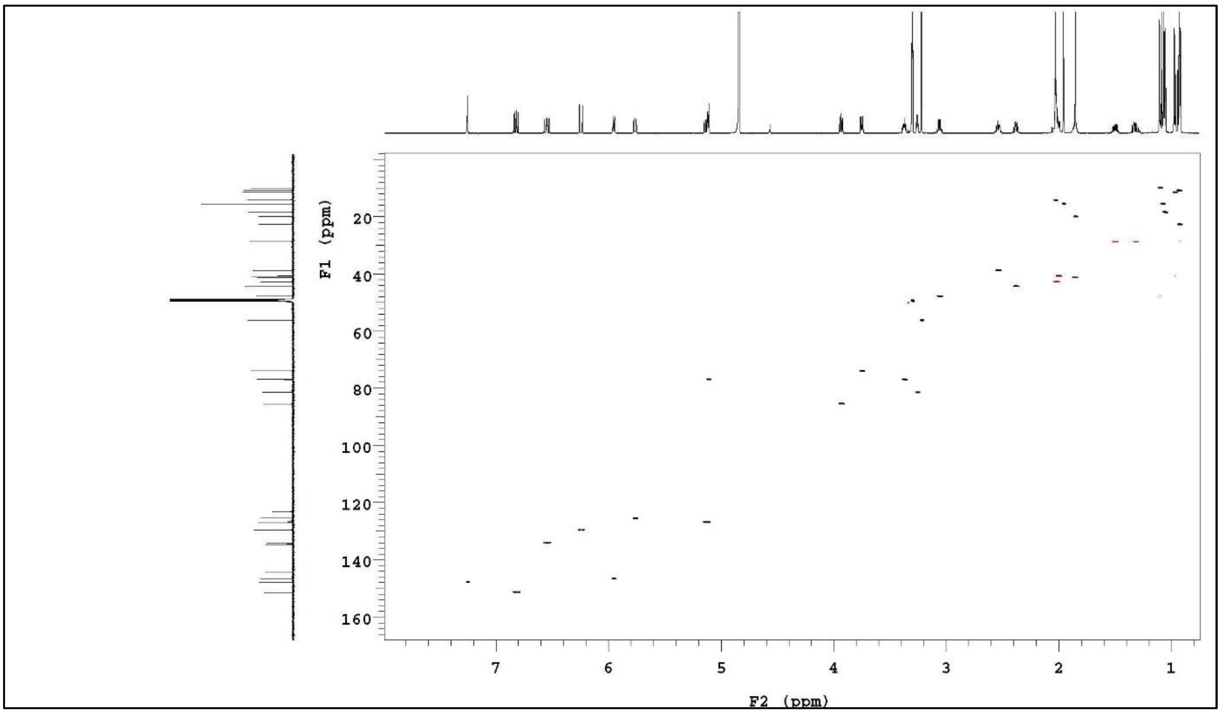

HMBC spectrum of oxohygrolidin (600 MHz, CD_3OD, 25 °C).

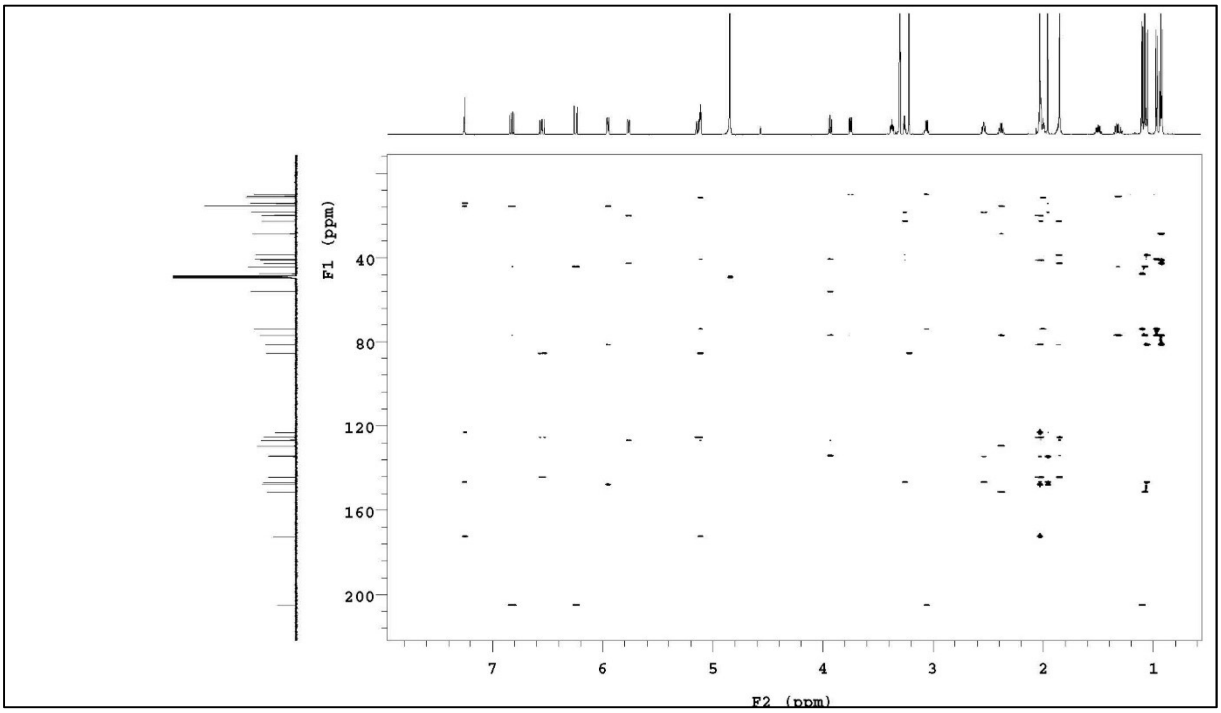

References

1. Gust, B. et al. λ Red-mediated genetic manipulation of antibiotic-producing *Streptomyces*. *Adv. Appl. Microbiol.* **54**, 107–128 (2004).
2. Makitrynskyy, R. et al. Pleiotropic regulatory genes *bldA*, *adpA* and *absB* are implicated in production of phosphoglycolipid antibiotic moenomycin. *Open Biol.* **3**, 130121 (2013).
3. Chater, K., F. & Wilde, L. C. *Streptomyces albus* G mutants defective in the *Sal*GI restriction-modification system. *J. Gen. Microbiol.* **116**, 323–334 (1980).
4. Myronovskyi, M., Welle, E., Fedorenko, V. & Luzhetskyy, A. Beta-glucuronidase as a sensitive and versatile reporter in actinomycetes. *Appl. Environ. Microbiol.* **77**, 5370–5383 (2011).
5. Fedoryshyn, M., Welle, E., Bechthold, A. & Luzhetskyy, A. Functional expression of the Cre recombinase in actinomycetes. *Appl. Microbiol. Biotechnol.* **78**, 1065–1070 (2008).
6. Bierman, M., Logan, R., O'Brien, K., Seno, E. T., Rao, R. N. & Schoner, B. E. Plasmid cloning vectors for the conjugal transfer of DNA from *Escherichia coli* to *Streptomyces* spp. *Gene* **116**, 43–49 (1992).
7. Muth, G., Nussbaumer, B., Wohlleben, W. & Pühler, A. A vector system with temperature-sensitive replication for gene disruption and mutational cloning in streptomycetes. *Mol. Gen. Genet.* **6**, 1–8 (1989).
8. Herrmann, S. et al. Site-specific recombination strategies for engineering actinomycete genomes. *Appl. Environ. Microbiol.* **78**, 1804–1812 (2012).
9. Hong, H. J., Hutchings, M. I., Hill, L. M. & Buttner, M. J. The role of the novel Fem protein VanK in vancomycin resistance in *Streptomyces coelicolor*. *J. Biol. Chem.* **280**, 13055–13061 (2005).
10. Tran, N. T., Den Hengst, C. D., Gomez-Escribano, J. P. & Buttner, M. J. Identification and characterization of CdgB, a diguanylate cyclase involved in developmental processes in *Streptomyces coelicolor*. *J. Bacteriol.* **193**, 3100–3108 (2011).
11. Sehin, Y. et al. Gene *ssfg_01967* (*miaB*) for tRNA modification influences morphogenesis and moenomycin biosynthesis in *Streptomyces ghanaensis* ATCC14672. *Microbiology* **165**, 233–245 (2019).
12. Kretschmer, A., Dorgerloh, M., Deeg, M. & Hagenmaier, H. The structures of novel insecticidal macrolides: bafilomycins D and E, and oxohygrolidin. *Agric. Biol. Chem.* **49**, 2509–2511 (1985).
